# In Vitro Evolution Reveals Noncationic Protein–RNA Interaction Mediated by Metal Ions

**DOI:** 10.1093/molbev/msac032

**Published:** 2022-02-08

**Authors:** Valerio G Giacobelli, Kosuke Fujishima, Martin Lepšík, Vyacheslav Tretyachenko, Tereza Kadavá, Mikhail Makarov, Lucie Bednárová, Petr Novák, Klára Hlouchová

**Affiliations:** 1 Department of Cell Biology, Faculty of Science, Charles University, BIOCEV, Prague, Czech Republic; 2 Earth-Life Science Institute, Tokyo Institute of Technology, Tokyo, Japan; 3 Graduate School of Media and Governance, Keio University, Fujisawa, Japan; 4 Institute of Organic Chemistry and Biochemistry, Czech Academy of Sciences, Prague, Czech Republic; 5 Department of Biochemistry, Faculty of Science, Charles University, Prague, Czech Republic; 6 Institute of Microbiology, Czech Academy of Sciences, Vestec, Czech Republic

**Keywords:** RNA–protein interaction, genetic code evolution, protein evolution, mRNA-display

## Abstract

RNA–peptide/protein interactions have been of utmost importance to life since its earliest forms, reaching even before the last universal common ancestor (LUCA). However, the ancient molecular mechanisms behind this key biological interaction remain enigmatic because extant RNA–protein interactions rely heavily on positively charged and aromatic amino acids that were absent (or heavily under-represented) in the early pre-LUCA evolutionary period. Here, an RNA-binding variant of the ribosomal uL11 C-terminal domain was selected from an approximately 10^10^ library of partially randomized sequences, all composed of ten prebiotically plausible canonical amino acids. The selected variant binds to the cognate RNA with a similar overall affinity although it is less structured in the unbound form than the wild-type protein domain. The variant complex association and dissociation are both slower than for the wild-type, implying different mechanistic processes involved. The profile of the wild-type and mutant complex stabilities along with molecular dynamics simulations uncovers qualitative differences in the interaction modes. In the absence of positively charged and aromatic residues, the mutant uL11 domain uses ion bridging (K^**+**^/Mg^2**+**^) interactions between the RNA sugar-phosphate backbone and glutamic acid residues as an alternative source of stabilization. This study presents experimental support to provide a new perspective on how early protein–RNA interactions evolved, where the lack of aromatic/basic residues may have been compensated by acidic residues plus metal ions.

## Introduction

Protein–RNA complexes are ubiquitous in modern life and are essential to many stages of the cell cycle and metabolism. The abundance of ribonucleoprotein complexes generally increases with organism complexity (Balcerak et al. 2019). However, it is thought that peptide–RNA interactions date to life’s origins and have changed substantially over time (Hsiao et al. 2009; Lupas and Alva 2017; Vázquez-Salazar and Lazcano 2018).

Some of today’s proteins and RNAs can act as independent functional and structural entities and their interaction is often transient. On the other hand, early oligopeptides and RNA are thought to have served as mutual cofactors and scaffolds, necessitating strong interactions (Lupas and Alva 2017; Vázquez-Salazar and Lazcano 2018). Structural analyses of today’s natural and artificially evolved protein–RNA interactions show that proteins build these interactions by exploiting positively charged and aromatic amino acids (Blanco et al. 2018; Srivastava et al. 2018). However, these amino acids (Lys, Arg, His, Phe, Tyr, Trp) were not likely available or extremely scarce in the prebiotic environment (Trifonov 2000; Higgs and Pudritz 2009; Granold et al. 2018). As a result, the specific nature of the interaction between early peptides and RNA remains an open question in the field.

Currently, there are two major hypotheses concerning the nature of early peptide-RNA binding. The first scenario holds that cationic amino acids were indispensable and their biosynthesis preceded the evolution of protein–RNA interaction (Blanco et al. 2018). Some studies suggest that the lack of canonical cationic amino acids in the early environment could be compensated by the presence of more abundant positively charged noncanonical amino acids originating from various prebiotic sources of organic material (Zaia et al. 2008; Cleaves 2010; Burton et al. 2012). Given that formation of oligopeptides preceded the emergence of ribosomal translation, it follows that their composition would have reflected the composition of the prebiotic broth including noncanonical cationic amino acids, such as 2,4-diaminobutyric acid or ornithine (Raggi et al. 2016; Vázquez-Salazar and Lazcano 2018; Longo et al. 2020). An alternative scenario of early peptide–RNA interaction proposes that the electrostatic interaction of RNA with peptides relied on anionic (Glu and Asp, both prebiotically abundant) rather than cationic amino acids. In this model, the interaction would be mediated via metal ions (Raggi et al. 2016; Vázquez-Salazar and Lazcano 2018). The two hypotheses are not mutually exclusive and may also represent different evolutionary stages or combinations of different interaction types over the evolutionary timeline. At the same time, there is a lack of experimental support for both of them.

To test whether strong protein–RNA interactions are conceivable in the absence of positively charged and aromatic amino acids, we performed a high-throughput variant analysis of a highly conserved RNA-binding domain of the ribosomal protein uL11. We designed combinatorial libraries that comprised: 1) the prebiotically plausible subset of 10 and 2) 14 “mid-stage” canonical amino acids, and selected RNA-binding mutants using mRNA display. Detailed characterization of viable variants from each of the two libraries unambiguously support that strong and specific protein–RNA interaction is possible in the absence of cationic and aromatic amino acids with the aid of metal ions. This mechanism of interaction confirms the feasibility of early RNA–protein interaction and brings attention to alternative RNA-protein engineering strategies.

## Results

### Design of uL11 RNA-Binding Domain Combinatorial Mutant Libraries

**Fig. 1.**
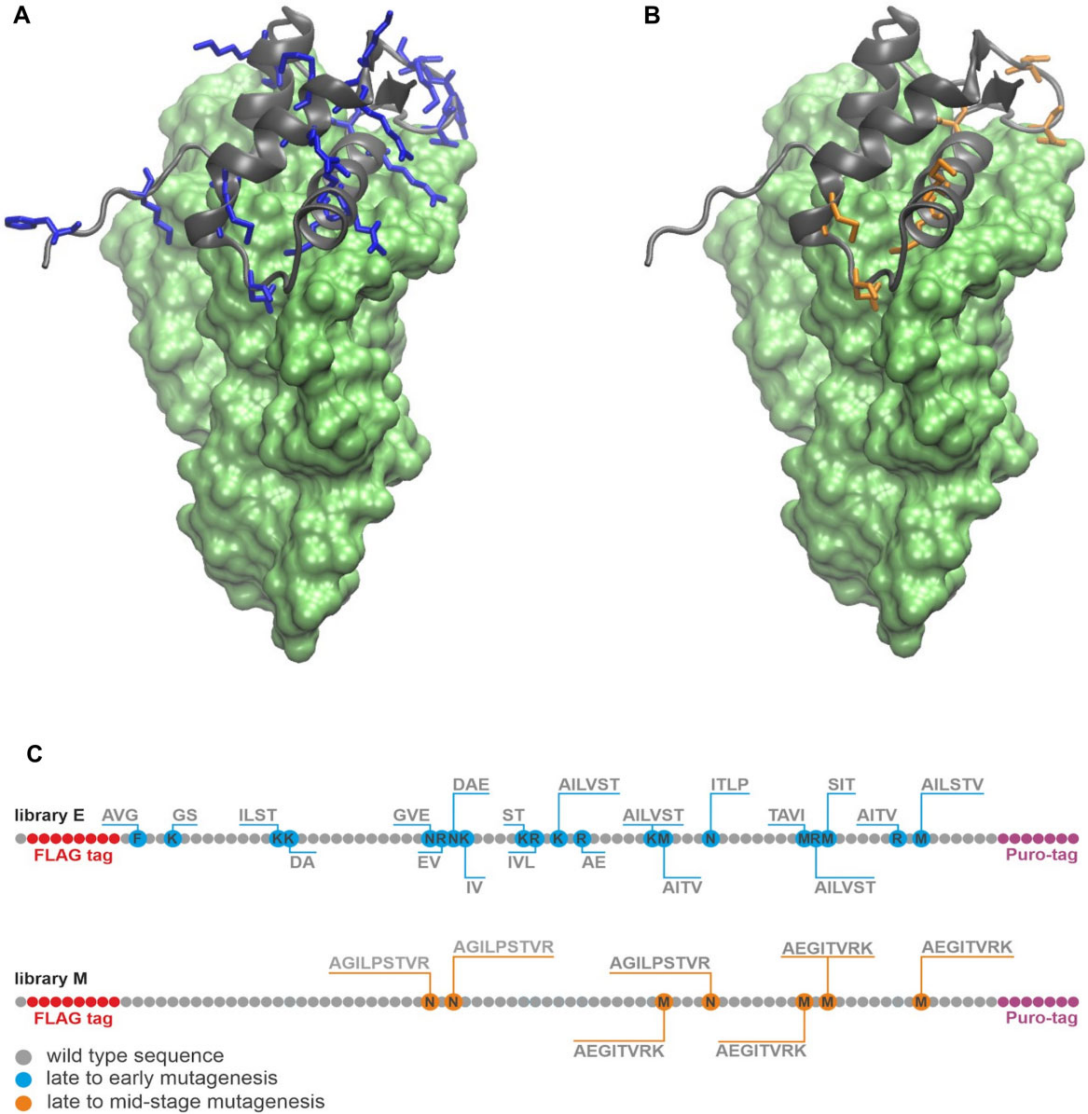
Design of CL11 variant libraries. Crystal structure of the CL11–58rRNA complex: (*A*) CL11: gray cartoon; CL11 substitution spots late to early amino acids: blue sticks; 58rRNA: green surface; (*B*) CL11: gray cartoon; CL11 substitution spots late to mid-stage amino acids: orange sticks; 58rRNA: green surface; (*C*) Randomization scheme for the CL11 -E and -M libraries.

To test the capacity of RNA–protein interaction in the absence of cationic and aromatic amino acids, we selected a highly conserved complex of the C-terminal RNA-binding domain of *Bacillus stearothermophilus* ribosomal protein uL11 (CL11) and a 58-nt domain of the large subunit ribosomal RNA (58rRNA) fragment as a selection target (fig. 1) (Conn et al. 2002). Two variant libraries were designed following the ranking of prebiotic amino acid composition of early genetic code, as derived by the meta-analysis of Higgs and Pudritz (2009). The “early” (E) library randomizes the native CL11 sequence by subsets of Gly, Ala, Asp, Glu, Val, Ser, Ile, Leu, Pro, Thr (fig. 1*A*), whereas the “mid-stage” (M) library includes all the wild-type cationic and aromatic amino acids (Lys, Phe, Arg, His) as visualized in figure 1*B*. Sequences in the E library are therefore composed exclusively of the “early” amino acids with 26% of the positions in the native sequence randomized (fig. 1*C*).

### mRNA Display of CL11 Combinatorial Libraries and RNA-Binding Selection

**Fig. 2.**
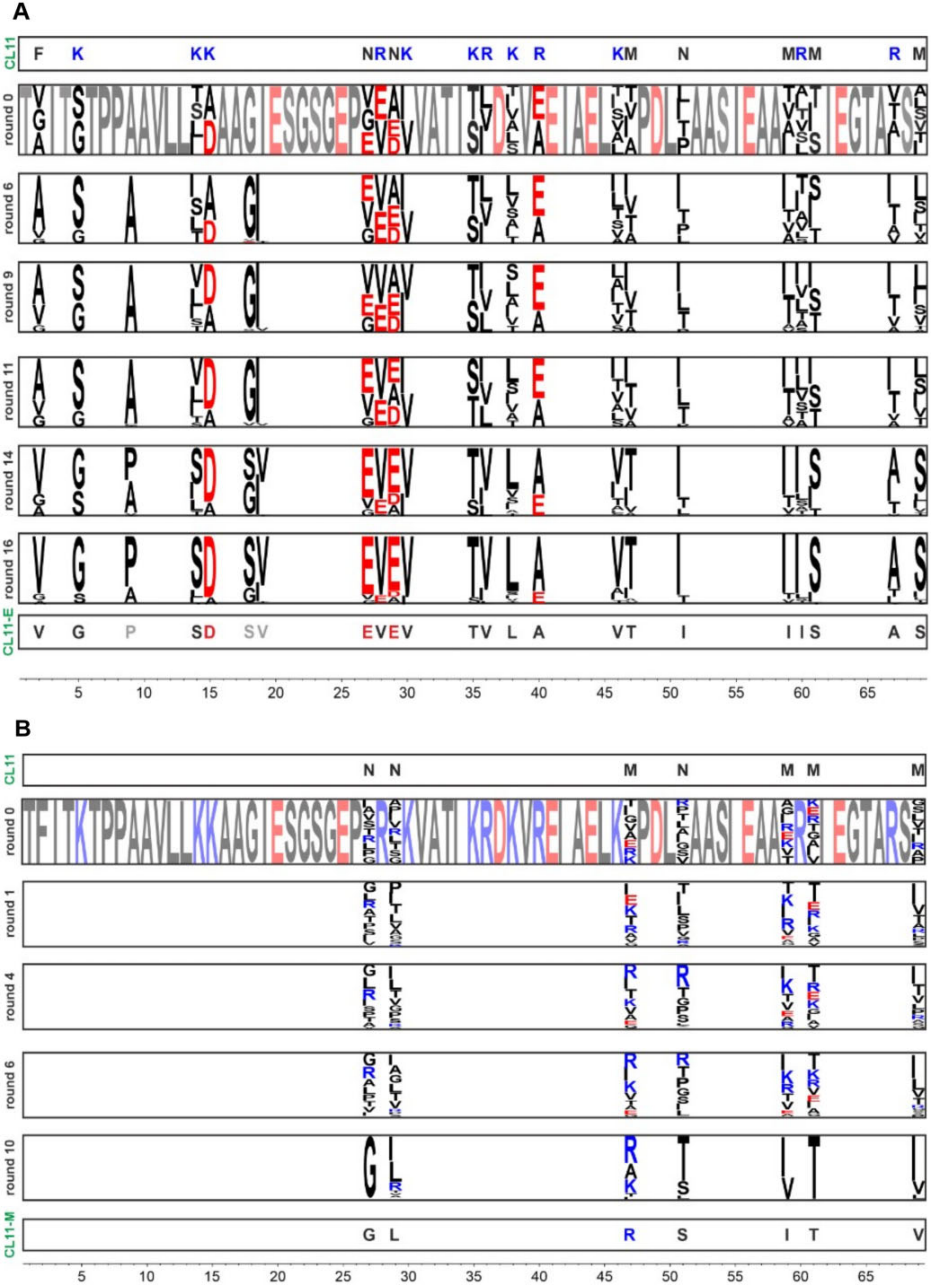
Selection of the CL11 variant libraries. The library E (*A*) and M (*B*) randomized fragment (1–69) sequencing results of the rounds 6, 9, 11, 14, and 16 and 1, 4, 6, and 10, respectively, displayed as sequence logos generated from 100 most abundant protein sequences in each selection round. The residue color code is based on amino acid charge (negatively charged: red, positively charged: blue and others: black).

The M and E oligonucleotide libraries were synthesized from two overlapping ssDNA oligonucleotides (see supplementary fig. S1*A*, Supplementary Material online). The oligonucleotide libraries were incorporated into a genotype–phenotype linked construct for mRNA display using a previously published protocol (supplementary fig. S1*B*, Supplementary Material online) (Reyes et al. 2021). Upon transcription and translation of the constructs, the best binding variants were selected against an immobilized 58rRNA, performing 10 and 16 rounds of the selections for libraries M and E, respectively (supplementary fig. S1*C* and *D*, Supplementary Material online; fig. 2). These numbers of mRNA display and selection rounds achieved distinct sequence consensus patterns at the randomized positions (fig. 2). Interestingly, the E library reached an acidic pattern at two regions that were of basic character in the wild-type sequence, specifically “SD” in place of “KK,” and an “EVEV” motif in place of “NRNK” (fig. 2*A*).

The most enriched sequences from each library (CL11-E and CL11-M, representing 4.28% and 9.17% of total read count, respectively) were selected for detailed characterization (supplementary fig. S2, Supplementary Material online). The most abundant sequence from the E library (CL11-E) contained three additional mutations at fixed amino acid positions in the original library (Asp9Pro, Gly18Ser, and Ile19Val). These mutations were likely introduced during the reverse transcription PCR step by the polymerases (fig. 2*A*).

### CL11, CL11-M, and CL11-E Structural and Binding Characterization

**Fig. 3.**
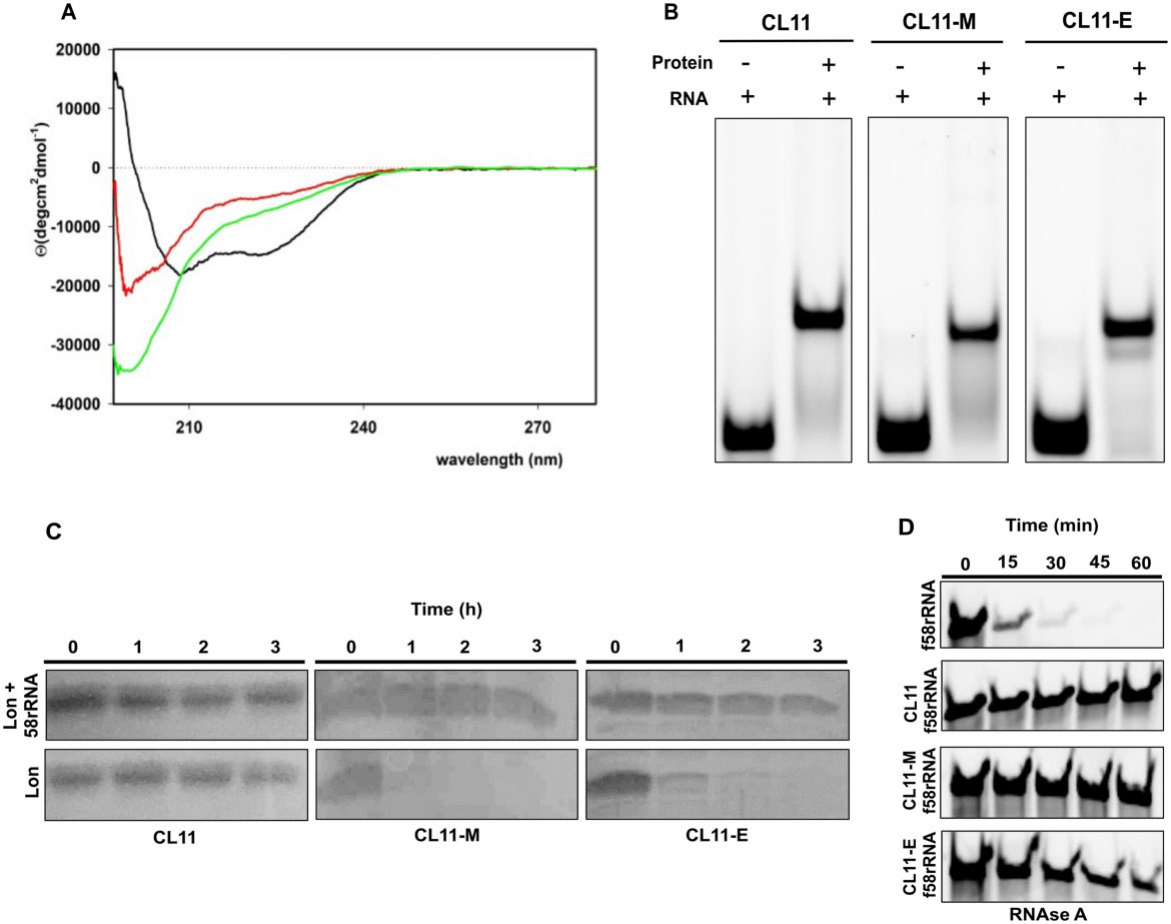
Characterization of CL11, CL11-M, and CL11-E variants and their binding to 58rRNA. (*A*) CD spectra of CL11 (black), CL11-M (red), and CL11-E (green) in buffer R. (*B*) EMSA assay where equimolar concentration of f58rRNA target was incubated with the different protein variants. Free f58rRNA was used as a negative control. (*C*) Proteolytic digestion of CL11, CL11-M, and CL11-E by Lon protease. The proteins were incubated with Lon in the presence or absence of 58rRNA in the reaction mixture. (*D*) f58rRNA digestion by RNase A in presence or absence of CL11, CL11-M, and CL11-E protein in the Lon buffer at 37°C.

Synthetic genes of the CL11 wild-type protein and CL11-E and CL11-M variants were expressed in *Escherichia coli* and purified to homogeneity. The protein samples were used to estimate the secondary structure content by circular dichroism (CD) (Uversky 2009). The CL11 wild-type spectra are indicative of high α-helical structure content (∼45%) by the minima at approximately 208 and 222 nm (fig. 3*A* and supplementary table S3, Supplementary Material online). In contrast, both the CL11-E and CL11-M variants have significantly less secondary structure content. Both mutants seem to be mostly disordered as indicated by a profound negative peak at 202 nm in the CD spectrum and with an isosbestic point at 211 nm in temperature dependent CD spectra (supplementary fig. S3*A*, Supplementary Material online).

The binding propensity of the purified proteins to 58rRNA was confirmed using the electrophoretic mobility shift assay (EMSA). The wild-type sequence (CL11), as well as the selected variants CL11-E and CL11-M, exhibited an electrophoretic shift on a native PAGE gel in the presence of fluorescent 58rRNA (f58rRNA) (fig. 3*B*). Dissociation constants of these binders were measured using surface plasmon resonance (SPR) (supplementary fig. S4*A*–*F*, Supplementary Material online). The *K*_D_ constant of the CL11-E variant is about one order of magnitude smaller than those of the CL11-M variant and the wild-type CL11 (2.2 × 10^−9^ M vs. 2.4 × 10^−8^ M and 2.5 × 10^−8^ M, respectively). The different *k*_on_ and *k*_off_ values indicate different kinetics of the RNA binding of these variants (table 1).

**Table 1.** Kinetic Parameters of CL11, CL11-M, and CL11-E Binding to 58rRNA Determined by SPR.

Protein	*k*_on_ (1/M×s)	*k*_off_ (1/s)	*K*_D_ (M) (*K*_off_/*K*_on_)
CL11	1.2 ± 0.4×10^4^	3.0 ± 0.2×10^−4^	2.5 ± 0.6×10^−8^
CL11-M	1.9 ± 0.9×10^4^	4.5 ± 0.9×10^−4^	2.4 ± 0.9×10^−8^
CL11-E	1.6 ± 0.8×10^3^	3.6 ± 0.8×10^−6^	2.2 ± 0.3×10^−9^

The SD (±) was calculated from two different sets of experiments each with three different protein concentrations.

The structural properties of the unbound versus 58rRNA-bound CL11 variants were inspected using mild Lon protease and RNase A treatment (fig. 3*C* and *D*). Although the wild-type CL11 is resistant to proteolysis in both free and RNA-complexed form, both CL11-E and -M variants are prone to proteolysis in the free forms. However, when complexed with 58rRNA, both mutant variants are resistant to proteolysis like the wild-type CL11 under the tested conditions (fig. 3*C*). Similarly, 58rRNA is significantly more resistant to RNase A degradation when complexed with the CL11 variants (fig. 3*D*).

**Fig. 4.**
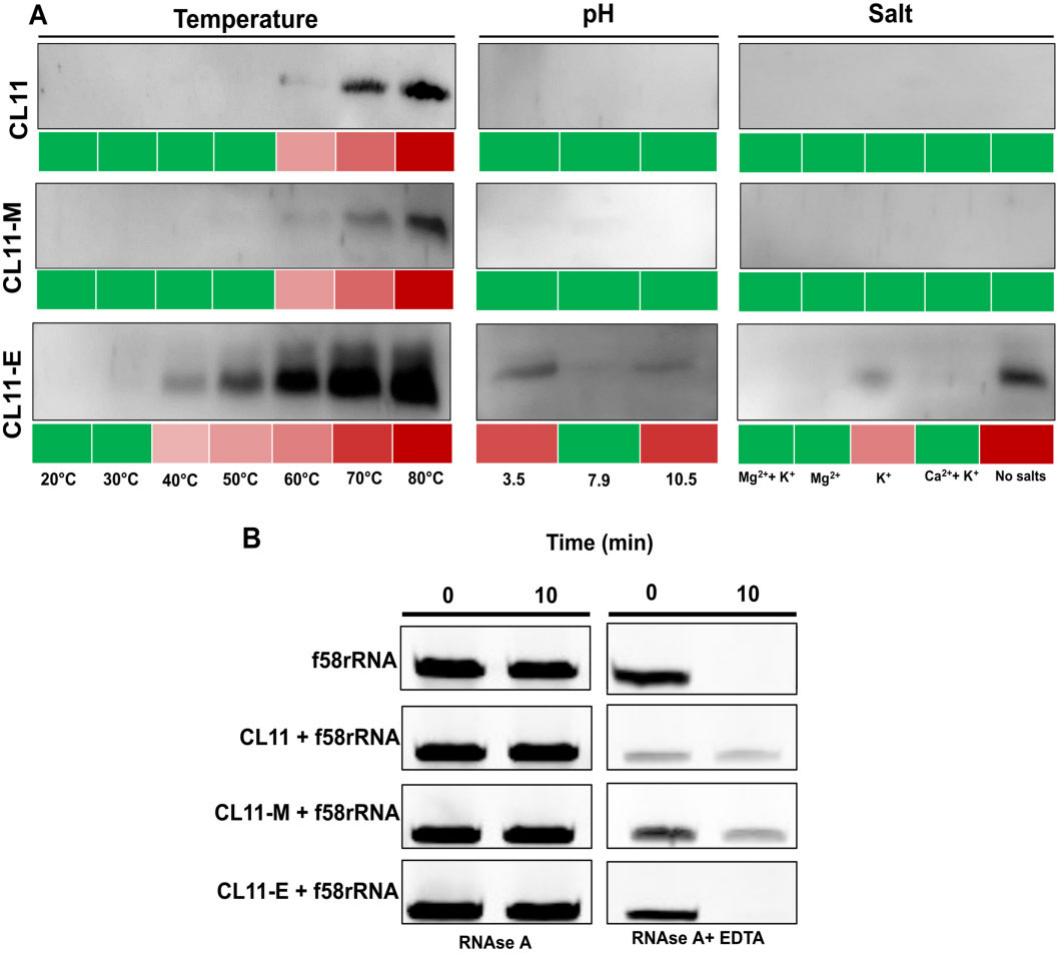
Characterization of the complex stabilities. (*A*) Protein–RNA complex stability at different temperatures, pH, and salt conditions. Green: no protein is detected from Western blot; increasing red intensity: increasing amount of protein detected on Western blot of the pull-down flow-through fraction (for uncropped Western blots and fractions remaining bound to the beads, see supplementary fig. S4*G*, Supplementary Material online). (*B*) f58rRNA digestion by RNase A in presence or absence of CL11, CL11-M, and CL11-E protein in the buffer R with and without 20 mM EDTA at 30°C.

To test the stability of the protein–RNA complexes under different conditions, additional pull-down experiments were performed with the purified proteins against the immobilized 58rRNA, similar to the binding selection procedure during the mRNA-display protocol. The protein–RNA complexes were incubated at different temperatures, pH, and salt contents (fig. 4*A*). The elution samples, indicating the unstable protein fractions of the complexes, were analyzed by Western blot (fig. 4*A* and supplementary fig. S4*G*, Supplementary Material online). Although the complex stabilities are the same in all the tested conditions for CL11 and CL11-M, the CL11-E–RNA complex shows a lower thermostability (40–50°C), lower stability at extreme pH conditions and is significantly affected by lack of Mg^2**+**^ and K^**+**^ ions. To verify the effect of metal ion concentration on the CL11-E–RNA complex, all the variant complexes were exposed to mild EDTA treatment (fig. 4*B*). The RNA part of the complex is stable to RNase A degradation under the EDTA treatment when complexed with the wild-type and CL11-M variant but gets degraded in the complex with CL11-E. In addition, the CD spectra of the uncomplexed proteins were collected at the same pH and “no salts” condition to resolve the potential reasons of the complex instability (supplementary fig. S3*B*, Supplementary Material online). Although the binding characteristics of the CL11-M mutant seem to be generally preserved with respect to the wild-type, the structural and RNA-binding properties of the CL11-E mutant prompted a further investigation to characterize the intriguing nature of this interaction.

We reasoned it would be difficult or impossible to crystallize the CL11-E–58rRNA complex; hence, an ion mobility mass spectrometry experiment was performed to characterize its overall structural properties. All CL11, CL11-E, and CL11-M were incubated with 58rRNA and each protein and protein–RNA complex was transferred to an electrospray compatible buffer for ion-mobility measurements. Unlike the CL11 wild-type apoprotein, the -E and -M variants exhibit a broader signal suggesting less ordered and multiple conformations (supplementary fig. S5*A*, Supplementary Material online). The collision cross-sections were calculated 2,100 Å^2^ for CL11–58rRNA, 2,050 and 1,900 Å^2^ for CL11-E–58rRNA, and 2,080 and 1,920 Å^2^ for CL11-M–58rRNA (supplementary fig. S5*B*, Supplementary Material online). We can conclude that all the complex particles kept a similar shape in solution.

### CL11 and CL11-E Computational Structural Analysis

**Fig. 5.**
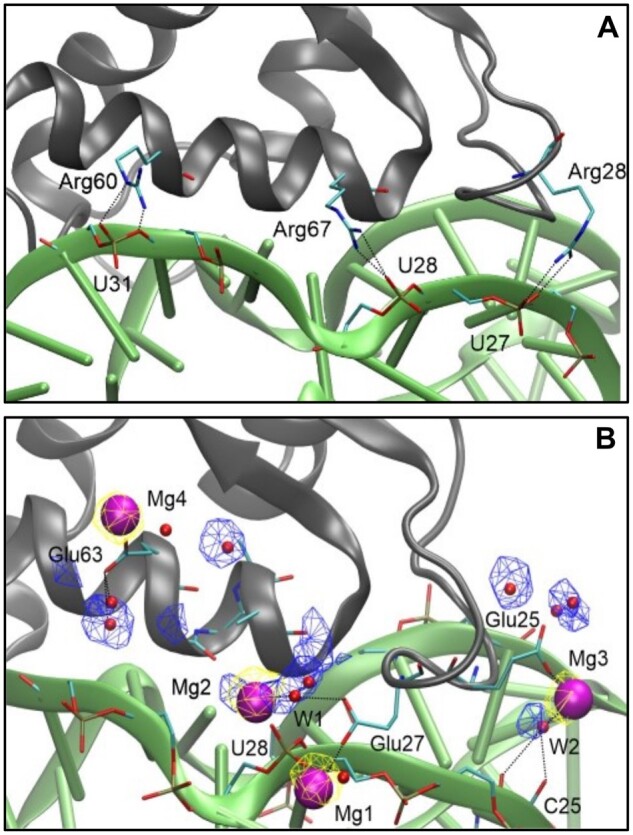
Molecular details of RNA–protein interaction. Representative snapshots from the last 500 ns of MD simulations showing the CL11/CL11-E proteins (gray cartoon) with important residues in sticks (cyan: C, blue: N, red: O, golden: P, hydrogens not shown), 58rRNA (green cartoon) with important residues in sticks, Mg^2+^ ions (purple spheres), and water molecules (red spheres). Yellow and blue meshes show the conserved sites for Mg^2+^ and water, respectively, at 0.2 and 0.25 occupancy isovalue. Black dotted lines indicate H-bonding or metal coordination. (*A*) CL11–58eRNA, (*B*) CL11-E–58rRNA.

Using the previously published X-ray structure of the CL11–58rRNA complex (PDB code 1HC8), an initial model for CL11-E was prepared by generating the appropriate mutations in PyMol. The wild-type and the CL11-E complexes with 58rRNA were prepared for molecular dynamics (MD) simulations to compare the RNA–protein interface interactions. Throughout 2 µs MD, the behavior of the proteins was relatively stable with average root-mean-square deviations of protein backbone atoms in secondary structure elements (three α-helices and two parallel β-sheets) of 0.6 and 1.1 Å, for CL11–58rRNA and CL11-E–58rRNA complexes, respectively. There were minor rearrangements around 1.3 µs and in the first 500 ns of the CL11–58rRNA and CL11-E–58rRNA complexes, respectively (supplementary fig. S6, Supplementary Material online). Nevertheless, the last 500 ns were stable in MD of both the systems studied and were thus used in further analyses. To investigate the nature of the CL11 versus CL11-E interaction with the 58rRNA, direct hydrogen bonds (H-bonds) were measured. 30 direct protein-RNA H-bonds (with an average occupancy of 63%) versus 17 (with an average occupancy of 53%) were recorded for CL11 and CL11-E with the 58rRNA, respectively (supplementary table S4, Supplementary Material online); that is, CL11 makes nearly twice as many direct H-bonds with the RNA as compared with CL11-E. In 70% of cases, CL11 forms H-bonds via protein side chains (12 in total from Arg28, Arg60, and Arg67, fig. 5*A*), whereas in the case of CL11-E, a slight majority (53%) of H-bonds are formed through backbone atoms. In addition, several water bridges were identified at the binding interface. In the case of CL11, these were mostly via Lys5, Lys14, Lys46, Arg 60, and Arg 67, whereas for CL11-E, the side chains of Ser21, Ser61, and Asp49 and the backbone of Ala10, Gly22, and Gly24 were the major residues involved (fig. 5*B*). Further, metal (K^+^/Mg^2+^) ion sites were observed to bridge the protein and the RNA but only in the simulation of the CL11-E–58rRNA complex. Four K^+^ ions were attracted to the areas of high electrostatic potential, that is, close to carboxylates of protein Glu side chains and to the phosphates of RNA backbone, but were in constant exchange between contact pairs and bulk solvent. Four presumed K^+^ sites were probed by manual replacement of K^+^ with Mg^2+^. Upon starting another 2 µs MD from this model, we observed stable occupation of these sites. Mg^2+^ ions were coordinated either directly by the 58rRNA (U28 in Mg1 site) or the protein side chains (Glu25, Glu27, or Glu 63 in Mg3, Mg1, and Mg4 sites, respectively) or indirectly via water bridges (W1, W2 in Mg2 and Mg3 sites, respectively) (fig. 5*B*). It should be noted that Glu27 is a mutation with respect to the wild-type CL11 and that Mg4 does not provide an interaction with the RNA. Taken together, based on the MD simulation data, we propose that three Mg^2+^ ions mediate unique CL11-E specific interactions with 58rRNA.

## Discussion

Our experiments show that protein binders of RNA can be found in sequences composed of a significantly reduced set of canonical amino acid alphabet. Using mRNA display, we selected rRNA binders from variant libraries of the ribosomal uL11 C-terminal domain that was composed of: 1) the prebiotically plausible subset “E” of 10 canonical amino acids (lacking both positively charged and aromatic residues) as well as 2) a reduced evolutionary mid-stage “M” alphabet of 14 amino acids (where all the positively charged and aromatic amino acids of the uL11 C-terminal domain protein sequence were conserved).

Extant RNA–protein interaction relies substantially on the presence of positively charged and aromatic amino acids (Blanco et al. 2018). However, these amino acids were heavily underrepresented in the early stages of life’s origins, whereas negatively charged amino acids were quite abundant (Trifonov 2000; Higgs and Pudritz 2009). Hence, our finding of a robust RNA–protein interaction in the absence of aromatic/basic residues may represent an early mode of intermacromolecular interaction under environmental conditions where these amino acids were sparse. The phenomenon of RNA–protein interaction is often considered central to early life and thought to predate last universal common ancestor (LUCA) and evolution of the full amino acid alphabet. Thus, the mechanism of how the early protein–RNA interaction could have been established in the absence of aromatic/basic residues has been debated. Besides a potential role of positively charged noncanonical amino acids (such as 2,4-diaminobutyric acid) that could be present in the prebiotic environment, a hypothesis of negatively charged amino acids interacting with RNA via metal ions (Mg^2+^ or Fe^2+^) has been raised (Raggi et al. 2016; Vázquez-Salazar and Lazcano 2018). Depending on the abundance of positive and negative amino acids in the prebiotic environment, both modes of interaction could be present while experimental data to support such hypothetical modes of interaction are lacking. Importantly, a tRNA-binding peptide composed of only Gly, Ala, Asp, and Val was recently selected using cDNA display but the structural mechanism of the binding has not been further characterized (Kumachi et al. 2016).

To shed more light on the possible mechanism of early protein–RNA interaction, we expressed the most enriched variants from the E and M pools of the CL11 protein domain and characterized them in both free and RNA bound forms. Interestingly, both CL11-M and -E variants are less structurally ordered than the wild-type protein in their free form as documented by their CD spectra and limited proteolysis profiles (fig. 3*A* and *C*). This is probably caused by the lowered number of internal stabilizing interactions which in the case of the -E variant may mainly be due to a complete lack of aromatic and cationic amino acids (Longo et al. 2015; Makarov et al. 2021). Nevertheless, both CL11 variants bind to the cognate 58rRNA with an overall similar affinity (*K*_D_ in the order of 10^−8^–10^−9^ M), even though the different kinetics (in case of CL11-E) of the binding suggests additional folding of the proteins upon binding and/or a different mode of interaction. Additional folding is further supported by the proteolytic resistance of the protein and 58rRNA species when complexed, in contrast with their proteolytic susceptibility in the free forms in case of the -M and -E variants (fig. 3*C* and *D*). In case of the CL11-E variant, the binding kinetics are significantly slower when compared with the wild-type CL11, suggesting that while the interaction is difficult to form, it is also more persistent.

However, the CL11-E–58rRNA complex stability is more sensitive to physicochemical conditions, such as temperature and pH. This feature was not recorded for the CL11-M–58rRNA complex which expressed an identical stability profile as the wild-type complex (fig. 4*A* and supplementary fig. S4*G*, Supplementary Material online), suggesting that the binding mode is consistent when CL11 variants have all the positively charged and aromatic amino acid residues conserved. Most significantly, the CL11-E–58rRNA complex becomes unstable when salt is removed from the buffer composition, yet the absence of salt does not affect the secondary structure properties of the protein (supplementary fig. S3*B*, Supplementary Material online). The complex is also destabilized upon addition of a mild chelator concentration under which the CL11 and CL11-M–58rRNA complexes remain stable (fig. 4*B*). This finding provides a hint that K^+^/Mg^2+^ ions may be important for the formation of the CL11-E–58rRNA complex, whereas the overall structural shape of the complex is preserved with respect to the wild-type as suggested by the complex native ion mobility experiment. We further compared the CL11 and CL11-E–58rRNA-binding properties using MD simulations. 30 direct H-bond interactions have been identified at the interface of wild-type CL11 and the 58rRNA, 12 of them mediated by Arg side chains. The latter interactions were obviously absent from the CL11-E binding interface. Further, water-mediated H-bonds via Lys side chains in CL11 were functionally compensated in CL11-E by water-mediated H-bonds via Ser side chains and Gly backbones. A unique feature of the CL11-E–58rRNA complex was the presence of K^+^/Mg^2+^ ion bridges found between acidic Glu side chains and the 58rRNA phosphate backbone, which indicates the possibility of a new compensatory interaction mode between the protein and 58rRNA (fig. 5). Although the MD simulation results are in line with the experimental observation of the CL11-E–58rRNA complex instability under no salt (no bridging cation) and low pH condition (protonated glutamic acid residues have a significantly reduced possibility to form the cation bridge interaction), the atomistic details of the binding interface should not be overinterpreted. Further investigations with MD as well as NMR, which are beyond the extent of this study, would shed more light on the studied interaction (Campagne et al. 2019). Previously, tens-of-microsecond-long or enhanced-sampling MD simulations proved useful in studying RNA–protein interfaces, including H-bonding, water, and ion bridges (Krepl et al. 2017; Bochicchio et al. 2018). Additionally, free-energy simulation methods may help reveal the affinity contributions of individual protein residue mutations in RNA–protein complexes (Krepl et al. 2017; Bochicchio et al. 2018). But still, force-field parameters for RNA may be problematic in some cases and are under constant development via comparison with quantum mechanics calculations (Bochicchio et al. 2018; Pokorná et al. 2018).

To the best of our knowledge, this is the first experimental indication of cation mediated RNA–protein interaction, obtained via an in vitro evolution approach. Proteins lacking positively charged and aromatic residues may represent features of early proteins that could have existed during the pre-LUCA period. The structural and functional properties of early proteins are still debated. Although previous studies have pointed out that acidic proteins can be soluble and sustain secondary structure information, it has generally been unclear how those proteins can form compact architectures and/or interact with the highly acidic nucleic acids (Doi et al. 2011; Tanaka et al. 2011; Longo et al. 2013; Shibue et al. 2018; Newton et al. 2019; Solis 2019). We have recently recorded a similar structural propensity of random sequence libraries formed by the full and early amino acids alphabets (lacking positively charged and aromatic residues) when inspected by limited proteolysis in cell-like environments (Tretyachenko et al. 2021). A study by the Tawfik group suggested that polyamines and divalent cations may have played an important role in promoting the folding of such early proteins (Despotović et al. 2020). Similarly, magnesium and potassium cations clearly play a special role in the ribosomal structure, a molecular fossil that reports on the earliest interactions between RNA and proteins (Petrov et al. 2011; Rozov et al. 2019). In the ribosomal central and most conserved region, magnesium ions have even been observed to mediate RNA–protein (protein uL2) interaction via water molecules (Petrov et al. 2012). More recently, RNA has been found to be generally stabilized by amino acid-chelated Mg^2+^, implying possibly a very fundamental role of metal ions in the coevolution of the RNA–protein world (Yamagami et al. 2018). Finally, our study suggests that such a mechanism of metal-ion assisted interaction could have enabled early RNA–protein interaction in the environment where positively charged amino acids were sparse. Although further examples are needed, our case study suggests the possibility that positively charged amino acids were added later to the protein alphabet to fine-tuning of RNA–protein transient interactions but were not explicitly necessary either.

## Materials and Methods

### Design of uL11 RNA-Binding Domain Combinatorial Mutant Libraries

Two variant libraries of the C-terminal RNA-binding domain of *Bacillus stearothermophilus* ribosomal protein uL11 (CL11) were designed as follows. The E library is composed only of the “early” amino acids (Gly, Ala, Asp, Glu, Val, Ser, Ile, Leu, Pro, Thr), whereas the M library includes “mid-stage” amino acids in addition (Lys, Phe, Arg, His). All the remaining residues in the CL11 sequence have been randomized for the above listed amino acids using the following logic. The substitution pools at each position were selected based on: 1) environment-specific amino acid substitution tables that have been developed on the basis of acceptability of mutations within folded proteins by comparative analysis of homologous proteins (Koehl and Levitt 1999), and 2) multiple alignments of the protein family. This approach limited the size of the variant libraries E and M to approximately 10^10^ and 10^6^, respectively. To represent all the desired substitution combinations in the E and M combinatorial libraries, degenerate codon DNA sequences for the library synthesis were designed using the SwiftLib tool (Jacobs et al. 2015).

### Electrophoretic Mobility Shift Assay

Protein–RNA complexes were analyzed following Xing et al. protocol (Xing and Draper 1995). Fluorescently labeled 58rRNA (f58rRNA) target was synthesized by Integrated DNA Technologies (Supplementary Material online). Purified proteins were incubated in 1:1 molar ratio with f58rRNA target (2 µM concentration of both) in buffer R (30 mM Tris, 20 mM MgCl_2_, and 175 mM KCl at pH 7.9) for 1 h at room temperature. Gels were visualized by fluorescence in Typhoon FLA 9500.

### Surface Plasmon Resonance Measurements

All SPR measurements were performed on a four-channel SPR sensor platform (PLASMON IV) developed at the Institute of Photonics and Electronics (IPE) of the Academy of Sciences of the Czech Republic, Prague. Gold SPR chips were functionalized following the Neburkova et al. protocol (Neburkova et al. 2018). Afterwards, 500 µl of 0.3 µM biotinylated target 58rRNA in buffer R + 0.1% Triton X-100 solution was loaded on the functionalized chip. Assay with immobilized neutravidin without 58rRNA target served as negative control to all kinetic experiments. CL11 (525 to 66 nM), CL11-M (200 to 21.25 nM), and CL11-E (11 to 2.7 µM) in buffer R + 0.1% Triton X-100 were injected (association phase) for several minutes, and then buffer R + 0.1% Triton X-100 alone was injected (dissociation phase). Obtained data were fitted by the logistic equation using TraceDrawer (Ridgeview Instruments AB), and *k*_on_, *k*_off_, and *K*_D_ values were calculated from Kinetic evaluation using OneToOne fitting model.

### Lon Protease Degradation Assays

Lon protease was expressed and purified according to Niwa et al. 2019 protocol (Niwa et al. 2019). CL11, CL11-M, and CL11-E (1.5 µM) were incubated with or without 58rRNA (30 µM) in Lon buffer (50 mM Tris–HCl, 10 mM MgCl_2_, 2 mM ATP, pH 8.35). After 1 h incubation at room temperature, Lon protease (266 µM) was added to the reaction. Each condition was carried out at 37°C for 3 h with aliquots withdrawn every 60 min. Reaction products were analyzed by 16% SDS–PAGE.

### RNAse A Degradation Assay

f58rRNA (1.5 µM) was incubated with or without CL11 (10 µM), CL11-M (10 µM), and CL11-E (80 µM) in buffer R with 20 mM EDTA or Lon buffer. After 2 h incubation at room temperature, commercial RNAse A (NEB) (3.3 ng/ml) was added to the reaction. Each condition was carried out at 30 or 37°C for 1 h with aliquots withdrawn every 15 min. The reactions were stopped by adding 10 µg of Proteinase K (NEB) and incubated for 5 min at 37°C. Reaction products were analyzed on 10% Mini-PROTEAN TBE-Urea Gel (BIO-RAD). Gels were visualized by fluorescence in Typhoon FLA 9500.

### Temperature, pH, and Salts Stability

Magnetic Dynabeads MyOne Streptavidin C1 (Invitrogen) and Magnetic Dynabeads M-280 Tosylactivated (Invitrogen) functionalized with Neutravidin (Invitrogen) were used to immobilize 1 μM of biotinylated 58rRNA according to the manufacturer’s recommendation. The functionalized beads were washed and then incubated for 1 h with 1 μM solution of different protein variants in Buffer R + 0.05% Triton X-100 at room temperature under gentle rotation. 20 μl (5 mg/ml) beads retaining the formed complex were washed and resuspended in buffer R + 0.05% Triton X-100 at the same initial concentration and incubated for 10 min at different ranges of temperature (20–80°C). In the pH stability experiment, the beads (5 mg/ml) were incubated for 1 h at room temperature at buffers with three different pH values: 50 mM glycine, 175 mM KCl, 20 mM MgCl_2_, 0.05% Triton X-100, pH 3.5 or 10.5, and buffer R. In the salt stability experiment, 5 mg/ml of functionalized beads were incubated for 1 h at room temperature in: buffer R, buffer R without KCl, buffer R without MgCl_2_, buffer R with 40 mM CaCl_2_ instead of MgCl_2_ and buffer R without KCl and MgCl_2_. Elution samples were loaded on 16% SDS–PAGE gel and analyzed by Western blot using Monoclonal Anti-6X His tag antibody produced in mouse conjugated with HorseRadish Peroxidase, HRP (Sigma), detected with a Immobilon Forte Western HRP substrate (Merck) and visualized by Amersham Imager 600.

### Molecular Dynamics Simulation

Starting from CL11–58rRNA complex (PBD: 1HC8, chains A, C) (Conn et al. 2002), the CL11-E–58rRNA complex was modeled by replacing the amino acids in question in PyMol, version 1.7.4 (The PyMOL Molecular Graphics System, Version 1.7.4, Schrödinger, LLC). Ions important for the structural stability of the RNA were retained in the model. These were: 1) all Mg^2+^ ions except two (residues 1165, 1166), 2) other two Mg^2+^ ions which were put in place of two Os^3+^ for computational simplicity, and 3) one K^+^ ion. Water molecules were added extending 12 Å from the solute. Mg^2+^ and Cl^−^ ions were added to a final concentration of 20 mM and further K^+^ and Cl^−^ ions were added to a final concentration of 175 mM. Mg^2+^ parameters of Allnér et al. (2012) were used. The systems were stepwise minimized, heated to 300 K and at a pressure of 1 atm in NpT ensemble, production runs of MD ensued for 2 μs. Frames were saved every 1 ns. MD topologies and trajectories can be accessed from Lepsik 2021 (Lepsik 2021). Occupancies of water and ion sites were analyzed using the VOLMAP tool. To explore the stability of K^+^ versus Mg^2+^ ions in the sites, we manually exchanged four K^+^ ions in the sites with four Mg^2+^ and started a new unrestrained MD run. The SHAKE algorithm was used to restrain all bond vibrations and hydrogen mass repartitioning to 3 Da allowed us to apply a time step of 4 fs. All the analyses were done in the CPPTRAJ program (Shitov et al. 1984).

## Supplementary Material

Supplementary data are available at *Molecular Biology and Evolution* online.

## Supplementary Material

msac032_Supplementary_Data
