# Supplementary material for: In Vitro Evolution Reveals Noncationic Protein–RNA Interaction Mediated by Metal Ions
Source: Mol Biol Evol. 2022 Feb 8;39(3):msac032. doi: 10.1093/molbev/msac032 (PMC8892947; doi:10.1093/molbev/msac032)
Supplement: msac032_Supplementary_Data [file msac032_supplementary_data.zip › Giacobelli_response.pdf]

November 30, 2021

Dear members of MBE Editorial Board,

We would like to thank you and the three reviewers for their critical reading of our manuscript and thoughtful remarks. In particular, we really appreciated the suggestions for additional experiments that we had not thought of ourselves, carried them out and believe that they further support our conclusions.

We proceed with a detailed response to each of the reviewers' comments. The original reviewers' comments are compiled below in **black**. Our responses are in blue.

Sincerely,

Klara Hlouchova

Reviewer: 1

Comments to the Author
This article describes the search for variants of ribosomal protein L11 C-terminal domain that use a simplified alphabet of 10 prebiotically plausible amino acids yet retain RNA-binding activity. The deeply surprising result is that such variants could be identified from a library in which the L11-C sequence had been mutagenized with subsets of the 10 allowed amino acids at all positions carrying other amino acids than one of the 10 (CL11-E). The authors seem to also have been unsure whether this could work, since they also performed the experiment with variants containing 14 amino acid types, including positive and aromatic side-chains (CL11-M). The main message (and importance) of the article is that protein binders of RNA can be found in sequences composed of a significantly reduced, prebiotically plausible set of canonical amino acid alphabet that is missing the main amino acids mediating the interaction with nucleic acids in present-day proteins. This is an astonishing result.

Beyond this finding, a lot of aspects relating to the actual mechanism employed by these variants to bind RNA remain uncertain. The data shown in the paper are compatible with CL11-E and CL11-M being largely unstructured in solution and folding to the wt structure on the RNA, but it remains conceivable that one or both proteins employ different geometries of interaction than the wt. Molecular dynamics simulations do not help to judge on this possibility, since the wt structure is assumed as a starting condition. (It therefore seems to me that the claim that "this is the first experimental demonstration of cation mediated RNA- protein interaction" is not really warranted at this point.)

I think that several experimental tests could provide important, albeit indirect support for the mode of interaction proposed here (the direct test would be by NMR, but I assume the authors do not have this option). One would be mild protease and nuclease treatment of the protein-RNA complexes as compared to the molecules alone. I would assume that CL11-E and CL11-M would be digested fully under conditions in which wt would not be degraded, but if they bind to RNA in the same geometry as wt, then I would expect a similar degradation profile for the three. The same could be true for the 58rRNA exposed to nuclease, which should yield a similar footprint for the three protein variants. The dependence on metal ions for the interaction of CL11-E could also be

explored further by titration with chelators. I do not find the western blots very convincing, given that CL11-E shows the worst binding in basic pH.

In conclusion, the finding that polypeptides using a simplified alphabet can bind RNA with comparable affinity to present-day ribosomal proteins is truly astonishing and a big step forward for the field. The mechanistic conclusions on the mode(s) of interaction are preliminary and will need to be worked out further, but quite possibly not in this manuscript.

First, we would like to express our gratitude to reviewer #1 for such a detailed and inspiring analysis of our work. We cannot but agree with the reviewer that it would be great to provide more experimental support for our conclusions and also that some of the mechanistic aspects of the interaction will await further research and confirmations using different model situations.
We also agree that it would be best to study the binding interface in more detail using NMR. However, we do not have this option due to the synthetic costs of the RNA. Nevertheless, we are really grateful for the suggestions about mild proteolysis analyses of the apo-proteins and complexes and the chelator titrations that we had not thought of before. We are happy to report that we performed the experiments (Fig. 3 C&D and Fig. 4B in the revised manuscript). We selected the Lon protease for this experiment based on its minimal bias towards different amino acid composition of the variants. As expected, the uncomplexed CL11-E and -M variants are more prone to cleavage than the wild-type variant, reflecting their less structured form in solution. At the same time, a similar degradation profile is observed for all three variants when complexed with RNA. This is true for both the protein and RNA parts (cleaved by RNAse A) of the complexes. In case of CL11-E, the degradation is slightly faster, probably caused by the lower k_{on} constant. In addition, the EDTA titration experiment supported the mechanism of CL11-E interaction with RNA via metal ions as RNA was promptly degraded under such conditions, unlike the CL11 and CL11-M complexes (Fig. 4B).
The following text was included in the Results and Discussion section of the update manuscript (along with the the description of the methodology).

Results:
The structural properties of the unbound vs. 58rRNA-bound CL11 variants were inspected using mild Lon protease and RNase A treatment (Fig. 3C&D). While the wild type CL11 is resistant to proteolysis in both free and RNA-complexed form, both CL11-E and -M variants are prone to proteolysis in the free forms. However, when complexed with 58rRNA, both mutant variants are resistant to proteolysis like the wild type CL11 under the tested conditions (Fig. 3C). Similarly, 58rRNA is significantly more resistant to RNase A degradation when complexed with the CL11 variants (Fig. 3D).

To verify the effect of metal ion concentration on the CL11-E-RNA complex, all the variant complexes were exposed to mild EDTA treatment (Fig. 4B). The RNA part of the complex is stable to RNase A degradation under the EDTA treatment when complexed with the wild type and CL11-M variant but gets degraded in the complex with CL11-E.

Discussion:
Interestingly, both CL11-M and -E variants are less structurally ordered than the wild-type protein in their free form as documented by their CD spectra and limited proteolysis profiles (Fig. 3A and 3C).

...the different kinetics (in case of CL11-E) of the binding suggests additional folding of the proteins upon binding and/or a different mode of interaction. This is further supported by the proteolytic resistance of the protein and 58rRNA species when complexed, in contrast with their proteolytic susceptibility in the free forms in case of the -M and -E variants (Fig. 3C and 3D).

Most significantly, the CL11-E-58rRNA complex becomes unstable when salt is removed from the buffer composition, yet the absence of salt does not affect the secondary structure properties of the protein (Figure S3). The complex is also destabilized upon addition of a mild chelator concentration under which the CL11 and CL11-M-58rRNA complexes remain stable (Fig. 4B).

We hope that inclusion of this additional data will satisfy the reviewer's comments and we are thankful for the suggestion of these experiments. In addition, we took the liberty and re-collected the ion mobility (IM-MS) spectra of all the uncomplexed and complexed variants (updated Supplementary Fig. S5). Unlike the CL11 wildtype apoprotein, the -E and -M variants exhibit a broader signal suggesting a less ordered and multiple conformations, respectively. The IM-MS analysis further indirectly supports the observation that the less ordered -E and -M variants probably undergo additional folding upon their binding to RNA. As concerns the modelling part, we do agree that it only gives a first "visualisation" of the structural mode of CL11-E/RNA binding which is compatible with the current experimental data but is definitely not the only one. We could envisage a standalone computational project employing specific force fields for intrinsically disordered proteins (see e.g. Curr Opin Struct Biol. 2018, 48: 40–48) and various enhanced sampling MD techniques (see e.g. J. Chem. Phys. 2019, 151, 070902) for the apo-proteins as well as simulating various encounter complexes by docking (e.g. Nature Protocols, 2010, 5, 883–897). We are confident that this highly interesting and at the same time challenging system will attract further attention.
In addition, the claim "this is the first experimental demonstration of cation mediated RNA-protein interaction" has been toned down to "this is the first experimental indication of cation mediated RNA-protein interaction".

Reviewer: 2

Comments to the Author
In this work, Giacobelli et al. use mRNA display to isolate an RNA-binding protein that does not contain aromatic or cationic amino acids, which are thought to have emerged later in the evolution of the genetic code. The high-affinity protein-RNA interaction was dependent on metal ion- and water-mediated interactions mediated by anionic residues, in contrast to modern RNA-binding proteins, which typically recognize RNA through direct interactions with cationic and aromatic amino acids. These results show that primordial RNA-binding proteins could have achieved high affinity for RNA even with a limited genetic code lacking aromatic and cationic amino acids and is a useful advance in our understanding of the evolution of primordial peptide-RNA interactions and the RNA world. The work is interesting, straightforward, and persuasive, and would be appreciated by the readership of Molecular Biology and Evolution. The main conclusions are basically supported by the data, although a couple of points would benefit from further experimental validation.

Major comments

1. The SPR data for CL11 and CL11-E (Fig. S4) is not very convincing, with poor fits to the data, only two or three analyte concentrations, and (seemingly) only a single experimental replicate. For CL11-E, although it is hard to read the axis labels, it also seems like the response is very low despite the high analyte concentration, possibly suggesting a large amount of inactive protein that would decrease the accuracy of the kinetic parameters. The finding that CL11 and CL11-E have a quantitatively similar affinity is an interesting point of the paper, so the SPR experiment should be replicated at least once to confirm this.

We agree with the reviewer that this data needed further validation. All the measurements were repeated at different and higher protein concentrations and the new K_d values were evaluated. The new graphs are included in Figures S4A-F.

2. The experiment in Fig. 3D and Fig. S4B appears to be lacking positive controls (e.g., total elution with SDS), making it impossible to interpret. For example, for CL11-E, bands are visible at pH extremes, but we don't know whether these represent a tiny fraction or the whole amount of protein loaded onto the beads. If positive controls were performed, they should be shown, and if they were not performed, the experiment should be repeated with positive controls. It should also be made clear which experiments were done side-by-side, e.g., through inclusion of the raw (uncropped) gel images in a Supplementary Data file. In particular, the observation here that RNA binding of CL11-E is dependent on metal ions is an important aspect of the paper and needs to be properly supported.

We are grateful to the Reviewer for considering the experiment in detail. The negative controls have been performed along with the main experiment and we are happy to include them (as well as the uncropped gel images of the main experiment data) in the Supplementary Figures. In fact, Reviewer #1 also raised concerns about this experiment. Based on the combined feedback, Fig. 3D (currently Fig. 4A) has now been re-designed to include the original data (concerning also Reviewer 2 minor comment no. 1). We agree with both reviewers that the metal ion bridging represents an important aspect of the paper and its significance has been further tested by including a chelator agent in the proteolysis assay suggested by Reviewer #1. The assay confirms that in presence of EDTA, the RNA is promptly cleaved by RNAse in case of the CL11-E complex, unlike in the case of CL11-M and the wt complexes. We hope that addition of this experiment in the results (Fig. 4B) will be perceived well by Reviewer 2.

3. While the CD experiments suggest that the structure of CL11-E is very different from CL11, the structure of CL11-E is basically unchanged throughout the MD simulations, even though the structural model is based on CL11. How do the authors explain this discrepancy? Does it mean that the simulated structure of CL11-E is not realistic? Although the simulation time is fairly short, I would expect an unstable structure to fall apart within a 2 μs simulation.

The Reviewer rightfully brings out the false tendency of protein force fields to form stable secondary structures. With the growing need to simulate intrinsically disordered proteins, fine-tuned specific force fields have recently been developed (see e.g. Curr Opin Struct Biol. 2018, 48, 40–48). But as these are not tested enough with nucleic acids, we did not opt to use them. In the simulations, we were primarily interested in the protein-RNA complexes, so we removed mentions about simulating apo-proteins from the Methods, Results and Fig. S6.
CD, IM-MS and newly added limited proteolysis assays (Fig. 3C) suggest that CL11-E is more disordered in the uncomplexed form.

4. Since the abundance of the metals was different at the origin of life it might be relevant to assess other metals or at least Fe2+ by electrophoretic shift on a native PAGE gel in presence of selected variants CL11-E and CL11-M and f58rRNA.

We agree with the reviewer that it would be great to get more insight about the interaction via different metal cations. However, Fe^{2+} belongs to very challenging metals to work with as it requires anoxic chamber operation (which we don't have access to). According to literature, the metal

cations that were selected in our study all belong to those relevant during the early stages of life's evolution and have been also selected based on their compatibility with the experimental assays. The assay presented in Fig. 4A was selected because of the EMSA low sensitivity and bad compatibility with metal salts.
That being said, we will be aiming for a follow-up study to address the complex binding mechanism in detail and will do our best to include a broader panel of metals if possible as we agree that this aspect is of great interest.

Minor comments

1. Fig. 3D should be replaced with Fig. S4B - it is better to show the actual data (i.e., the blots themselves) rather than a subjective summary.

We agree with the Reviewer that inclusion of the actual data would better satisfy the standards expected by the readership of MBE. The figure has now been replaced and is newly annotated as Fig. 4A.

2. p. 4, third paragraph. "The stability of these sites in MD was increased upon exchanging K+ with Mg2+". What does this mean? K+ entered the RNA-protein interface first and was later displaced by Mg2+? By what measure was the stability increased?

The Reviewer is right in that the explanation was unclear. We thus rephrased it and added sentences to Results and Methods.

Results
Four K^+ ions were attracted to the areas of high electrostatic potential, i.e. close to carboxylates of protein Glu side chains and to the phosphates of RNA backbone but were in constant exchange between contact pairs and in bulk solvent. Four presumed K+ sites were probed by manual replacement of K^+ with Mg^{2+}. Upon starting another 2 μs MD from this model, we observed stable occupation of these sites.

Methods
To explore the stability of K+ vs. Mg2+ ions in the sites, we manually exchanged four K+ ions in the sites with four Mg2+ and started a new unrestrained MD run.

3. Fig. 3C. The meaning of ± should be specified.
This has been changed in the manuscript.

4. Fig. 4. In panel B, according to the legend the mesh shows the conserved water and Mg2+ binding sites, but what does this mesh physically represent? Also, according to the legend the snapshots are from the last 500 ns of the simulation, but how were they selected from that 500 ns interval? Are they representative snapshots or final snapshots?

Again, we thank this reviewer for pointing these flaws out. The following information has been added to Fig. 4 caption:
"at 0.2 and 0.25 occupancy isovalue."
"Representative"

5. Fig. S3A. This panel is not really informative without a legend. Maybe just selecting a few curves for each protein would be a better option.

We are thankful to the reviewer for pointing this out. Along with the problematic font size, the legend to the figure has been added.

Minor corrections

1. Abstract: "...where the lack of aromatic/basic residues was compensated by acidic residues plus metal ions". "Was" should be replaced with "could have been", "may have been" etc.
This has been changed in the manuscript

2. p. 2, results paragraph 1: "includes all the wild-type cationic and aromatic amino acids". This sounds like all aromatic amino acids were included, so it should be changed to "some aromatic amino acids".
In fact, the -M variant contains all the aromatic amino acids of the wt as there are no aromatics in the CL11 beyond Phe. We have added the protein sequences to the Supplementary Information for better clarity.

3. p. 3, first paragraph. "(i) environment-specific amino acid substitution tables..." Not sure what this means - a reference would help.
This has been changed in the manuscript

4. p. 3, last paragraph. Text refers to Fig. 4C - should be Fig. 3C.
This has been changed in the manuscript

5. p. 3, last paragraph. "...the CL11-E-RNA complex is destabilized at lower temperatures..." This sounds like the complex is unstable at low temperatures and stable at high temperatures. It should be changed to "...the CL11-E-RNA complex shows lower thermal stability..." or similar.
This has been changed in the manuscript

6. p. 7, Molecular dynamics simulation. "Ions important for the structural stability..." I could not really understand this sentence. Is it correct? "Ions important for the structural stability of the RNA (all Mg2+ ions except residues 1165 and 1166), two Os3+ ions, which were replaced by Mg2+ for simplicity, and one K+ ion were retained in the model."

Rephrased to be better understood, it now reads:
"*Ions important for the structural stability of the RNA were retained in the model. These were: i) all Mg^{2+} ions except two (residue numbers 1165, 1166), ii) other two Mg^{2+} ions which were put in place of two Os^{3+} for computational simplicity and iii) one K^{+} ion.*"

7. Legend of Fig. 2 is incorrect (it is a duplicate of the Fig. 3 legend).
This has been changed in the manuscript

8. Some details in the figures are not legible: Fig. S3 axis labels, Fig. S4 axis labels, Fig. S5 insets, Table S2 text. Either the resolution or text size should be increased.

This has been changed in the manuscript

9. Cropping of the gels in Fig. S1C should be made explicit using a black line.
This has been changed in the manuscript

Reviewer: 3

Comments to the Author

The article by Giacobelli et al. addresses the RNA-Protein interaction using in vitro selection. The authors select L11 C-terminal domain and create a library within which they select for RNA-binding variant. The selected variant binds to RNA with a similar overall ability although it is less structured. The article conducts structural characterization of the RNA-Protein complexes formed using CD and the binding was characterized using EMSA and SPR. Robustness and stability of the complexes was tested by varying experimental conditions like temperature, pH and salt contents.

Major:
1) The ribosome is a molecular fossil containing a record of the primordial events and it is a perfect system to study early evolution. However, the examples of RNA-protein interactions mediated by metal cations described in this study do not appear to be primordial given that they are not present in conserved protein segments nor in ancestral sequence reconstructions. These examples were engineered to fit a model of a reduced alphabet. Specifically, the authors have not given arguments supporting the relevance of the selected RNA-protein pair to model early RNA-protein interactions in the absence of cationic amino acids. The C-terminal domain of L11 that was selected for this study is highly conserved in sequence across phylogeny, yet it lacks RNA-protein interactions mediated by metal cations.
This study confirms the feasibility of RNA-protein interactions in the absence of cationic amino acids. It seems to be a general mechanism that can occur in extant RNA-protein interactions as well as in any other moment of the history of coded proteins. While the findings of the study are intrinsically important for understanding interactions of non-cationic peptide and RNA. However, the study does not address the question stated in the title of the manuscript (i.e. a primordial RNA-Protein interaction mediated by metal cations). Therefore, I would suggest to restructure the manuscript around the idea of characterization the interactions between the bob-cationic peptides and RNA and to change the title accordingly.

We are thankful to the reviewer for this perspective. We agree that there is a lack of examples of the metal-mediated interactions preserved in today's biology as well as ASR-recovered sequences. Nevertheless, as we mention in the discussion of our manuscript:
"In the ribosomal central and most conserved region, magnesium cation has even been observed to mediate RNA-protein (protein L2) interaction via water molecules (Petrov et al. 2012)"
We would argue that the reason for the lack of this phenomenon in extant biology is that the stage of alphabet evolution (from the prebiotically plausible to the full canonical amino acid repertoire) that we mimic in our experimental setup precedes the time of LUCA. Hence it would not be in the resolution power of ASR and would be beyond something we can easily detect still in today's biology. We assume that once e.g. cationic amino acids would be incorporated into protein sequences, the metal mediated interaction would preferentially be supplemented. While this phenomenon clearly requires more mechanistic characterization, our impression is that the metal-

mediated interaction is not as “tunable” as the direct cationic amino acid - RNA interaction which would thus be of advantage for biological systems to evolve.
The term “primordial” was hence based purely on the lack of the late amino acid availability and we agree that it is not necessarily related to the specific target of our study. We also agree that the title of our study may thus be misleading and propose to rephrase it to:
“In vitro evolution reveals non-cationic protein - RNA interaction mediated by metal ions”

2) Furthermore, in the current study, the authors attempted to address two separate questions: a) the ability of a given peptide sequence to fold and b) its ability to interact with RNA (in a folded or non-folded forms).The structural aspects of the apo proteins and their RNA complexes are not described in sufficient detail. The authors noted that “Both mutants seem to be mostly disordered” Do they expect that either of them would form a folded structure? A molten globule?

We agree with the reviewer that the originally submitted manuscript included only very limited information about the structural properties of the apo-proteins and their RNA complexes as methods of detailed structural characterization (such as NMR and x-ray crystallography) were unfortunately not accessible for the study of the proteins and/or their complexes. Nevertheless, the CD spectra (Fig. 3A) implied that the -M and -E apo-protein variants contain less secondary structure content then the wt CL11. This observation is further supported in the revised manuscript by (i) a limited proteolysis experiment that was suggested by Reviewer 1, and (ii) ion mobility (IM-MS) spectra of all the uncomplexed and complexed variants. Briefly, the uncomplexed CL11-E and -M variants are more prone to cleavage than the wt variant, reflecting their less structured form in solution. At the same time, a similar degradation profile is observed for all three variants when complexed with RNA. This is true for both the protein and RNA parts (cleaved by RNAse A) of the complexes (currently Fig. 3 C&D). Similarly, the IM-MS spectra show that unlike the CL11 wildtype apoprotein, the -E and -M variants exhibit a broader signal suggesting a less ordered and multiple conformations, respectively (updated Supplementary Fig. S5).

3) The authors state that “the different kinetics of the binding suggests additional folding of the proteins upon binding and/or a different mode of interaction. In the case of the CL11-E variant, the binding kinetics are significantly slower” Do they observe any conformational change in the MD simulations? Our attempt to predice the fold of the CL11-E by AlphaFold2 did not result in a successful prediction of a folded domain. If the authors anticipate a conformational change of CL11-E variant upon its binding to RNA, can they list any evidence that it would fold into a globular structure?

We are really thankful to the reviewer for attempting AlphaFold2 prediction of CL11-E and sharing the outcome with us. We did not have access to this prior to our manuscript submission. However, we are not surprised by the lack of folding of the E apo-protein. Our hypothesis that there is additional folding of the CL11-E (and partly probably also CL11-M) was based on (i) the CD spectra of the apo-protein, showing less secondary structure content, (ii) the ion mobility experiment of the RNA-complexed wt and E variants showing their similar shape in solution, hence suggesting the same form upon binding, and (iii) the different binding kinetics. Thanks to the reviewer #1 suggestion about extending these analyses by limited proteolysis experiments, we can now add some more evidence (albeit still indirect) supporting our hypothesis. While both CL11-M and -E variants are more prone to proteolysis by the Lon protease (selected because of its lack of bias towards specific amino acids) when compared with the wt variant, both of these variants are similarly protease resistant when complexed with RNA (Fig. 3C). In addition, the mechanism of the -E variant binding to RNA via metal ions has been further supported by RNAse cleavage of the complexes in the presence of EDTA (Fig. 4B in the updated manuscript). While

the wt and -M complexes are stable under mild EDTA concentration, the RNA in the -E variant complex is promptly degraded under the same conditions.
To see the conformational changes in MD, we would need to use enhanced-sampling MD techniques (see e.g. J. Chem. Phys. 2019, 151, 070902), which is beyond the scope of the current study. We hope that a follow-up study that would concentrate on the mechanistic aspects of the studied interaction (and its other examples) will further resolve some of these issues.

4) Since no direct structural analysis is provided, the authors should provide the structural models from their MD simulations in the SI, and to compute the cross-sectional areas from their simulated complexes to compare those with the experimental data. Do experimental and MD studies point to the same conclusions?

Modeled PDB files of CL11 and CL11E complexes with 58rRNA and their MD topologies and trajectories are now made publicly available. A mention is added to the Methods:". MD topologies and trajectories can be accessed from ref.: Lepsik, Martin (2021), "Molecular Dynamics of CL11 and CL11-E complexes with 58rRNA", Mendeley Data, V2, doi: 10.17632/hsg96vsxdw.1
The ion mobility experiments on CL11-58rRNA and CL-11-E-58rRNA complexes concluded from collision cross sections that both complexes have a similar shape in solution. We arrive at a similar conclusion by comparing the protein RMSD of both complexes (0.6 and 1.1 A, respectively; see pg. 4 and Fig. S6)

Minor:

a) Parts of the Results section would be more suitable for the Methods section.
This has been changed in the manuscript. Redundant information in the Results section has been either deleted or moved to the respective parts of the Methodology.

b) "Today's proteins and RNAs can act as independent functional and structural entities". This is not true for all extant proteins or for all extant RNAs.
We agree with the reviewer and apologize for the negligence. The sentence has now been changed to read:
"*Some of today's proteins and RNAs can act as independent functional and structural entities*"

c) "This is probably caused by the lowered number of internal stabilizing interactions which in the case of the -E variant may mainly be due to a complete lack of aromatic amino acids (Longo et al. 2015; Makarov et al. 2021)." This does not seem to be true since the only aromatic residue replaced appears to be at position 2 (Figure 2A) of the C terminal domain of uL11.
We are thankful to reviewer #3 for pointing this out. This is indeed true. While one of the references included with this statement claimed that even one aromatic amino acid can bring a significant difference in protein stability, we agree with the reviewer that our conclusion here may be dubious. The sentence has been changed to say:
"*may mainly be due to a complete lack of aromatic and cationic amino acids*"

d) Ban's notation (PMID: 24524803) should be used for naming of the ribosomal proteins
This has been changed in the manuscript

e) The caption of Figure 2 appears to be incorrect
Figure 2. Characterization of CL11, CL11-M and CL11-E variants and their binding to 58rRNA. (A) CD spectra of CL11 (black), CL11-M (red) and CL11-E (green) in 30 mM Tris, 20 mM $MgCl_2$ and 175 mM KCl at pH 7.9. (B) EMSA assay where equimolar concentration of f58rRNA target was incubated with the different protein variants. Free f58rRNA was used as a negative control. (C) Kinetic parameters of CL11, CL11-M and CL11-E binding to 58rRNA determined by SPR. (D) Protein-RNA complex stability at different temperatures, pHs and salts presence. Green: no protein is detected from western blot; increasing red intensity: increasing amount of protein detected on Western blot of the pull-down flow-through fraction (for original Western blots see Fig S4B)
This has been changed in the manuscript
