# Supplementary material for: In Vitro Evolution Reveals Noncationic Protein–RNA Interaction Mediated by Metal Ions
Source: Mol Biol Evol. 2022 Feb 8;39(3):msac032. doi: 10.1093/molbev/msac032 (PMC8892947; doi:10.1093/molbev/msac032)
Supplement: msac032_Supplementary_Data [file msac032_supplementary_data.zip › Supplementary Material.pdf]

In vitro evolution reveals non-cationic protein-RNA interaction mediated by metal ions

Valerio G. Giacobelli, Kosuke Fujishima, Martin Lepšík, Vyacheslav Tretyachenko, Tereza Kadavá, Mikhail Makarov, Lucie Bednárová, Petr Novák, Klára Hlouchová*

*To whom correspondence may be addressed. Email: klara.hlouchova@natur.cuni.cz

Supplementary Information Text

Oligonucleotide sequences

>Forward E library
5`-
AAAAAGTGGCCTGTAATACGACTCACTATAGGGACACCAATAGAGAAAGAGGAGAAATACTAGATGGACTATAAA
GATGACGACGATAAGACCGBAATTACCRGCACCCCGCCGGCGGCGGTGCTGCTGWYAGMCGCGGCGGGCATTGAA
AGCGGCAGCGGCGAACCG-3`

>Reverse E library
5`-
TTTTTCACCTGATCCGCTGCCATCTTCCACCACAATGCCTRHGCTTRYCGCGGTGCCTTCAATGVTTRHTRYCGC
CGCTTCAATGCTCGCCGCTRKCAGATCCGGTRYTRHCAGTTCCGCAATTTCTKCCACTRHATCTABGSTAATGGT
CGCCACTAYKKCTWCTHCCGGTTCGCCGCTGCCGCTTTCAATGCCCGCC-3`

>Forward M library
5`-
AAAAAGTGGCCTGTAATACGACTCACTATAGGGACACCAATAGAGAAAGAGGAGAAATACTAGATGGACTATAAA
GATGACGACGATAAGACCTTTATTACCAAAACCCCGCCGGCGGCGGTGCTGCTGAAAAAAGCGGCGGGCATTGAA
AGCGGCAGCGGCGAA-3`

>Reverse M library
5`-
TTTTTCACCTGATCCGCTGCCATCTTCCACCACAATGCCGVBGCTGCGCGCGGTGCCTTCAATTNYGCGTNYCGC
CGCTTCAATGCTCGCCGCGVBCAGATCCGGTNYTTTCAGTTCCGCAATTTCGCGCACTTTATCGCGTTTAATGGT
CGCCACTTTGVBGCGGVBCGGTTCGCCGCTGCCGCTTTCAATGCCCGCC-3`

>RT-PCR Forward primer
5`-
AAAAAGTGGCCTGTAATACGACTCACTATAGGGACACCAATAGAGAAAGAGGAGAAATACTAGATGGACTATAAA
GATGACG-3

>RT-PCR Reverse primer
5′-TTTTTCACCTGATCCGCTGCC-3′

>Gateway Forward primer CL11-E
5`-
GGGGACAAGTTTGTACAAAAAAGCAGGCTTCAAAGAGGAGAAATACTAGATGCATCACCATCACCATCACACCGT
TATCACCGGTACCCCG-3`

>Gateway Forward primer CL11-M
5`-
GGGGACAAGTTTGTACAAAAAAGCAGGCTTCAAAGAGGAGAAATACTAGATGCATCACCATCACCATCACACATT
CATCACCAAGACACCC-3`

>Gateway Reverse primer CL11-E and CL11-M
5`-GGGGACCACTTTGTACAAGAAAGCTGGGTCTTTTTCACCTGATCCGCTGCCTTA-3`

>CL11 DNA sequence
5`-
ATGCATCACCATCACCATCACACCTTCATCACCAAAACCCCGCCGGCTGCTGTTCTGCTGAAAAAAGCTGCTGGT
ATCGAATCTGGTTCTGGTGAACCGAACCGTAACAAAGTTGCTACCATCAAACGTGACAAAGTTCGTGAAATCGCT
GAACTGAAAATGCCGGACCTGAACGCTGCTTCTATCGAAGCTGCTATGCGTATGATCGAAGGTACCGCTCGTTCT
ATGGGTATCGTTGTTGAAGACTAA-3`

>CL11-E DNA sequence

5`-
ATGCATCACCATCACCATCACACCGTTATCACCGGTACCCCGCCGCCGGCTGTTCTGCTGTCTGACGCTGCTTCT
GTTGAATCTGGTTCTGGTGAACCGGAAGTTGAAGTTGTTGCTACCATCACCGTTGACCTGGTTGCTGAAATCGCT
GAACTGGTTACCCCGGACCTGATCGCTGCTTCTATCGAAGCTGCTATCATCTCTATCGAAGGTACCGCTGCTTCT
TCTGGTATCGTTGTTGAAGACTAA-3`

>CL11-M DNA sequence

5`-
ATGCATCACCATCACCATCACACATTCATCACCAAGACACCCCCGGCGGCAGTTTTATTAAAAAAAGCTGCCGGC
ATTGAGTCAGGCAGCGGGGAACCTGGGCGTTTGAAAGTTGCTACGATTAAACGCGACAAGGTCCGTGAGATCGCT
GAACTGAAACGCCCTGACTTGTCAGCGGCTTCGATCGAGGCAGCGATCCGTACAATCGAAGGGACCGCCCGCAGC
GTAGGCATCGTTGTCGAAGATTAA-3`

Puromycin complementary sequence

5`-GGCAGCGGATCAGGTGAAAAA-3`

Puromycin tag fragment

5`-pCCCTTCACCTGATCCGCTGAAAAAAAAAAAAAAAAAA-(Spacer18)-(Spacer18)
(Fluorescein-dT)-(Spacer18)-CC-(Puromycin)-3`

Biotinylated 58rRNA target

5`-biotin-(TEG)-GCCAGGAUGUAGGCUUAGAAGCAGCCAUCAUUUAAAGAAAGCGUAAUAGCUCACUGGU-
3`

Fluorescent 58rRNA target

5`-fluorescein-(Spacer9)-
GCCAGGAUGUAGGCUUAGAAGCAGCCAUCAUUUAAAGAAAGCGUAAUAGCUCACUGGU-3.

Protein sequences

>CL11 sequence

MTFITKTPPAAVLLKKAAGIESGSGEPNRNKVATIKRDKVREIAELKMPDLNAASIEAAMRMIEGTARSMGIVVE
D

>CL11-M sequence

MTFITKTPPAAVLLKKAAGIESGSGEPGRLKVATIKRDKVREIAELKRPDLSAASIEAAIRTIEGTARSVGIVVE
D

>CL11-E sequence

MTVITGTPPPAVLLSDAASVESGSGEPEVEVVATITVDLVAEIAELVTPDLIAASIEAAIISIEGTAASSGIVVE
D

Supplementary methods

mRNA display of CL11 combinatorial libraries and selection of RNA binders. Each library M and E were prepared by Klenow extension of two overlapping single-stranded DNA oligonucleotides, including all the necessary sequence motifs for transcription and addition of the FLAG tag to the N'-terminal part of the protein product (Supplementary Fig.S1A). ssDNA oligonucleotides were synthesized and PAGE purified by Integrated DNA Technologies. Annealing was performed by heating complementary oligonucleotide mixture (2 µM final concentration of each) in NEB1 buffer provided with 200 µM dNTPs to 90ºC for 2 minutes and cooling down to 25ºC for 10 minutes. Annealed product was filled in by Klenow extension to form double-strand DNA (dsDNA) as follows: 10 U of Klenow polymerase (NEB) was added to annealed oligonucleotides, incubated for 5 minutes at 25°C, 37°C for 1 hour and at 50°C for 15 minutes. Final dsDNA libraries were further column purified using the DNA Clean and Concentrator kit (Zymo Research). In the following transcription, 1µg of DNA library was used as a template for mRNA synthesis by AmpliScribe T7 Flash Transcription kit (Lucigen). mRNA was purified by NucleoSpin® RNA Clean-up Kit (Macherey-Nagel) and used for ligation with puromycin tagged oligonucleotide fragment synthesized by Integrated DNA Technologies *via* T4 RNA ligase reaction (for details see Reyes et al. 2021). Ligated products were purified (final yield ~30%) and used for translation of protein-mRNA conjugated library, following the mRNA display protocol (Figure S1D) (Reyes et al. 2021). To minimize false positives, 300 to 400 ng mRNA-protein conjugates were mixed with pre-equilibrated magnetic Dynabeads MyOne Streptavidin C1 (Invitrogen) in buffer R (30 mM Tris, 20 mM $MgCl_2$ and 175 mM KCl at pH 7.9) at 25°C with gentle mixing for 40 minutes, twice. The unbound products in the flow-through were collected and incubated for 1 hour at room temperature with 200 nM of the biotinylated 58rRNA target synthesized by Integrated DNA Technologies, Inc. The binding step was followed by biotinylated 58rRNA immobilization with bound protein library to 4.5 µL of pre-equilibrated streptavidin magnetic beads in buffer R and then washed 3-8 times with 500 µL of buffer R + 0.1% Triton X-100. Selected variants were eluted by heating the streptavidin affinity matrix for 2

minutes at 90°C in 5 µl of RNase free water. Total elution volume was used as a template for reverse transcription reaction (OneTaq® One-Step RT-PCR Kit, NEB) using previously described methods (Reyes et al. 2021). Reverse transcribed cDNA served as a template for the following selection rounds. mRNA display was carried out for 16 rounds for E library and 10 rounds for M library. Enriched DNA pools from selected rounds were submitted to next-generation sequencing via the Illumina MiSeq platform.

Sequencing and data analysis. Rounds 6, 9, 11, 14 and 16 of library E selection and rounds 1, 4 and 10 of library M were analyzed by high throughput sequencing on Illumina MiSeq. Prior to sequencing library preparation, quantification was carried out on Quantus™ Fluorometer (Promega). Total 100 ng of DNA sample was used as an input for the library preparation by NEBNext Ultra II DNA Library Prep kit (NEB) with AMPure XP purification beads (Beckman Coulter). Length of the prepared library was determined by Agilent 2100 Bioanalyzer (Agilent Technologies), quantified by Quantus Fluorometer (Promega). Sample was sequenced on MiSeq Illumina platform using the Miseq Reagent Kit v2 500-cycles (2x250) in a paired-end mode. Raw data was processed with Galaxy platform and sequence analysis of assembled and filtered paired reads was performed with MatLab scripts developed at Heinis lab (Rebollo et al. 2014; Afgan et al. 2018)**.**

Cloning, expression and purification of CL11, CL11-M and CL11-E. The CL11, CL11-M and CL11-E genes were synthesized with N-terminal 6X Histidine tag by Invitrogen and amplified by PCR and cloned into expression vector pDEST14 (Invitrogen) *via* Gateway cloning system (Gateway Technology). The cloned genes were used to transform OverExpress C41(DE3) pLysS chemically competent *E. coli* cells (Invitrogen). Fresh transformants were suspended in 10 ml of sterilized ampicillin-supplemented (100 µg/ml) LB-medium and grown overnight at 37ºC and 220 rpm. 2 ml of the pre-culture were inoculated into 2 L of sterile LB-medium supplemented with ampicillin (100 µg/ml) and grown in a rotary incubator (Innova 4300, New Brunswick Scientific) at 37ºC and 220 rpm. Expression was induced after OD_{600} reached a value of ~0.9 by addition of IPTG to a final concentration of 1 mM. Cells were cultivated for further 3.5 hours at 25ºC and 200 rpm and harvested by centrifugation (6000 rpm, 10 minutes, 4ºC). Pellets were resuspended in B-PER Bacterial Protein Extraction Reagent (Thermo Fisher Scientific) at 2.5 mL/g wet cells pellet with 1.5 U/mL of RNase A and DNAse I (Jena Bioscience) and EASYpack protease inhibitor cocktail (Sigma). Cell suspension was incubated at room temperature for 20 minutes on a vascular device at 60 rpm. Cells were centrifuged at 18,000 rpm for 20 minutes. The supernatants were loaded on 1 mL of Talon Metal Affinity Resin (Clontech Laboratories, Inc.) equilibrated with Talon buffer A (50 mM NaH_2PO_4, 50 mM of glutamic acid potassium salt, 300 mM NaCl, pH 7.2) and incubated under agitation for 30 minutes at room temperature. Column was washed with Talon buffer B (300 mM NaCl, 50 mM NaH_2PO_4, 50 mM of glutamic acid POTASSIUM salt, 5 mM imidazole, pH 7.2). Samples were eluted by 2 mL of Talon buffer C (300 mM NaCl, 50 mM NaH_2PO_4, 50 mM of glutamic acid potassium salt, 250 mM imidazole, pH 7.2). The CL11 and CL11-M fractions were dialyzed and loaded on HiTrap CaptoS 5 mL cation exchange column (GE Healthcare) equilibrated with buffer A (10 mM NaH_2PO_4 and 2 mM KCl at pH 7). The column was washed with five column volumes of buffer A. Bound proteins were eluted with 0-50% gradient of buffer B (10 mM NaH_2PO_4, 2 M NaCl and 2 mM KCl at pH 7). Fractions containing proteins of interest were collected, concentrated and loaded on Superdex 75 10/300 gel filtration column (GE Healthcare) equilibrated in a buffer containing 30 mM Tris, 20 mM $MgCl_2$ and 175 mM KCl at pH 7.9. CL11 and CL11-M fractions were collected, concentrated, dialyzed in buffer R and stored at -80ºC. CL11-E eluate from Talon affinity purification was collected and incubated in Talon buffer A with 7 M urea for 16 hours at room temperature. The sample was loaded on 0.5 mL of Talon Metal Affinity Resin equilibrated with Talon buffer A + 7M urea and incubated for 2 hours at room temperature. Column was washed with Talon buffer B + 7M urea. Sample was eluted by 0.650 mL of Talon buffer C + 7M urea. The eluted sample was loaded on HiLoad 16/600 Superdex 75 gel filtration column (GE Healthcare) equilibrated in a buffer R. Fractions containing CL11-E were collected, concentrated, dialyzed in buffer R and stored at -80 ºC. Protein concentrations were determined by amino acid analysis using a Biochrom 30+Series Amino Acid Analyser (Biochrom, UK).

Circular Dichroism. The ECD spectra were measured using the Jasco 1500 spectropolarimeter equipped with the Peltier holder PTC-517 in the far-UV (195-280 nm) spectral range. The ECD spectra were collected in different buffers composition (50 mM glycine, 175 mM KCl, 20 mM $MgCl_2$, pH 3.5 or 10.5, buffer R and buffer R without KCl and $MgCl_2$) and in temperature range 5 °C to 95 °C with the

step of 5 °C in 0.2 mm path length quartz cell (scanning speed of 10 nm/min, response time of 8 seconds, scanning step 0.5 nm) and with a sample concentration of 0.2 mg/mL. After baseline correction, the final spectra were expressed as molar ellipticity (*q*) ($deg.cm^2.dmol^{-1}$) per residue. The numerical analysis of secondary structure was performed using the CDPro software package (Sreerama and Woody 2000; Sreerama and Woody 2004).

Native ion mobility - mass spectrometry (IM-MS) measurements. Native IM-MS experiments were conducted on Waters Synapt G2Si instrument equipped with traveling wave IMS (TWIMS). The complex was assembled mixing 27 µL of 30 µM CL11, CL11-E or CL11-M sample with 3 µL 300 µM RNA in buffer R. Immediately prior MS analysis, samples were buffer exchanged to ammonium acetate (150mM, pH 6.9) using Zeba™ Micro Spin Desalting Columns (7K MWCO, 75 µL, Thermo Fisher Scientific). Desalted samples (5µM protein or 25µM protein-58rRNA complexes) were loaded into quartz nESI emitters pulled on P-2000 laser pullers (Sutter instruments). Measurement was performed in positive ion mode for mass range 500-5000 *m/z* with IMS wave velocity 750 m/s and height 30 V. For sample ionization ESI voltage (0.5-1 kV) was applied to platinum wire placed into nESI emitter containing sample solution. Obtained data were analyzed using MassLynx 4.1 (Waters) and OriginPro2015 (OriginLab). Collision cross sections (CCS) evaluation was performed using logarithmic calibration on native proteins (Cyt C, UBQ and bLG) (Bush et al. 2010; Allen et al. 2016).

SI References

Afgan E, Baker D, Batut B, Van Den Beek M, Bouvier D, Ech M, Chilton J, Clements D, Coraor N, Grüning BA, et al. 2018. The Galaxy platform for accessible, reproducible and collaborative biomedical analyses: 2018 update. *Nucleic Acids Res.* 46:W537–W544.

Allen SJ, Giles K, Gilbert T, Bush MF. 2016. Ion mobility mass spectrometry of peptide, protein, and protein complex ions using a radio-frequency confining drift cell. *Analyst* 141:884–891.

Bush MF, Hall Z, Giles K, Hoyes J, Robinson C V., Ruotolo BT. 2010. Collision cross sections of proteins and their complexes: A calibration framework and database for gas-phase structural biology. *Anal. Chem.* 82:9557–9565.

Rebollo IR, Sabisz M, Baeriswyl V, Heinis C. 2014. Identification of target-binding peptide motifs by high-throughput sequencing of phage-selected peptides. *Nucleic Acids Res.* 42.

Reyes SG, Kuruma Y, Fujimi M, Yamazaki M, Eto S, Nishikawa S, Tamaki S, Kobayashi A, Mizuuchi R, Rothschild L, et al. 2021. PURE mRNA display and cDNA display provide rapid detection of core epitope motif via high-throughput sequencing. *Biotechnol. Bioeng.* 118:1736–1749.

Sreerama N, Woody RW. 2000. Estimation of protein secondary structure from circular dichroism spectra: Comparison of CONTIN, SELCON, and CDSSTR methods with an expanded reference set. *Anal. Biochem.* 287:252–260.

Sreerama N, Woody RW. 2004. Computation and Analysis of Protein Circular Dichroism Spectra. *Methods Enzymol.* 383:318–351.

Figures and Tables

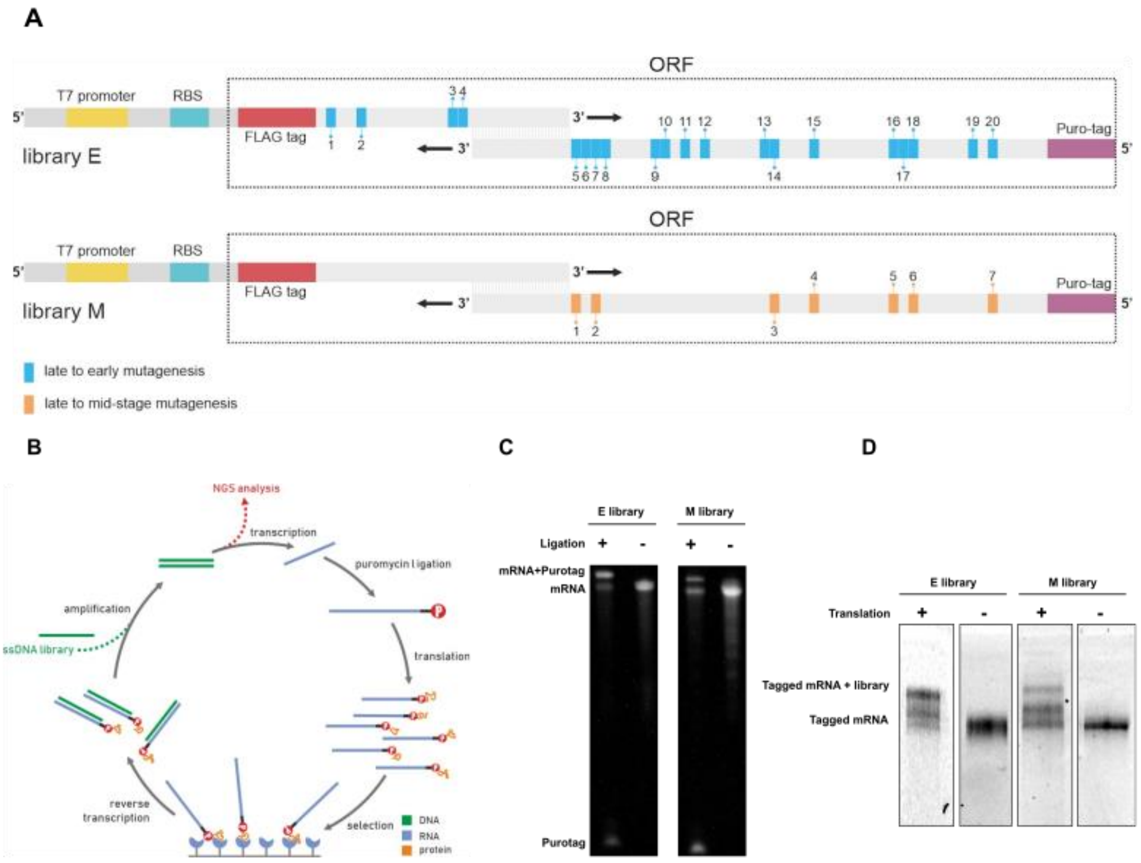

Fig. S1. mRNA display and preparation of the library E and M. (A) Design of the ssDNA libraries expression cassette (RBS - ribosomal binding site, ORF - open reading frame). (B) Schematic representation of the mRNA display and selection pipeline. The methodology starts with the annealing and the amplification of the designed ssDNA library. The DNA library is in vitro transcribed to mRNA library and undergoes ligation with the puromycin-DNA tag. Tagged product is translated and the resulting sample is mRNA-protein conjugate used for the selection process toward 58rRNA target. The selected variant conjugates are reverse transcribed and sequenced. (C) Images of mRNA ligation with Purotag fragment for M and E library resolved in 8 % polyacrylamide TBE with 8 M Urea gels with GelRed staining. (D) Images of the translated tagged mRNA resolved in 8 % polyacrylamide TBE with 8 M Urea gels with fluorescent visualization.

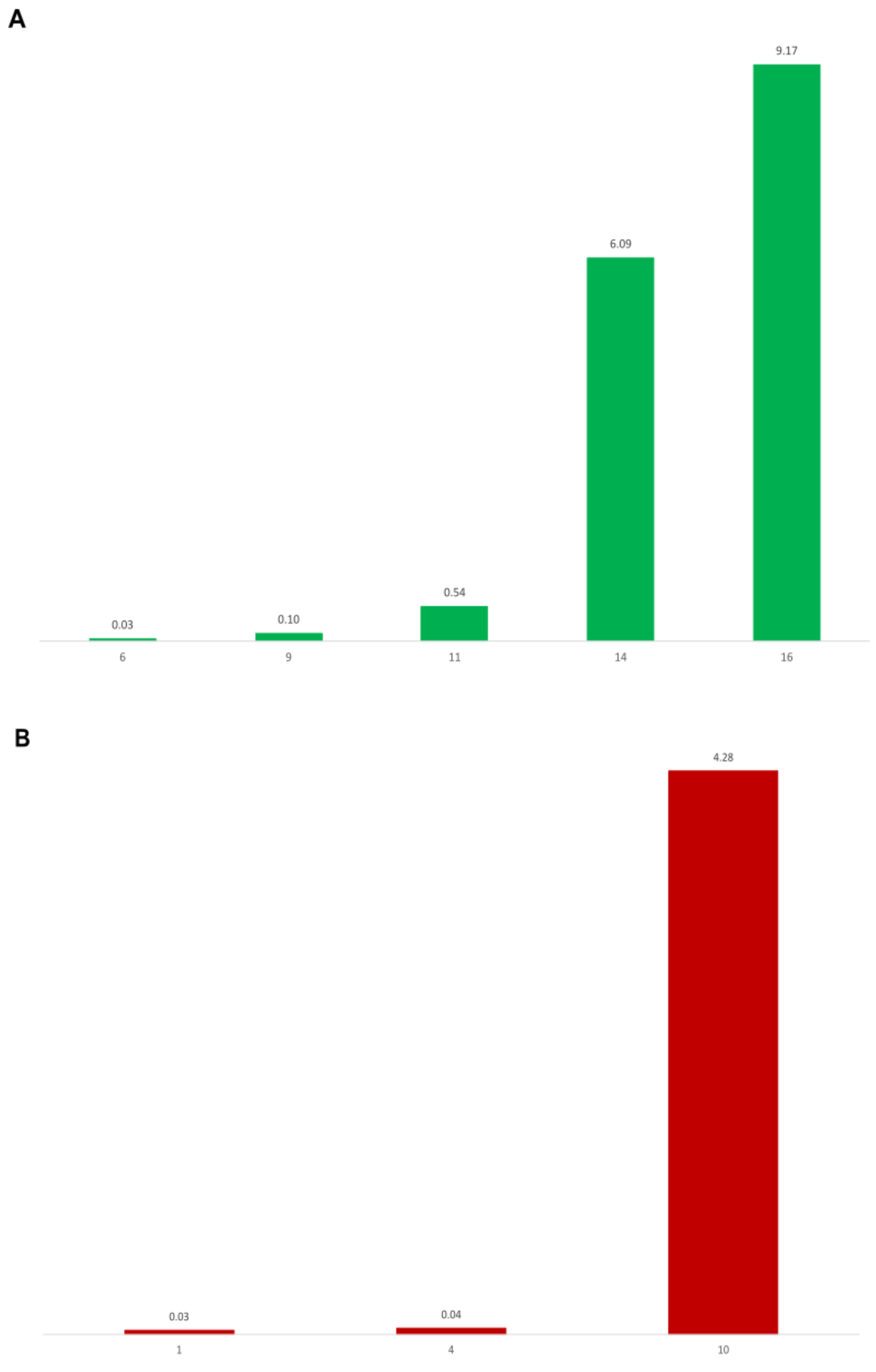

Fig. S2. Analysis of the mRNA display selection sequencing data (A) CL11-E enrichments from round 6th to 16th round selection. (B) CL11-M enrichments from round 1st to 10th round selection.

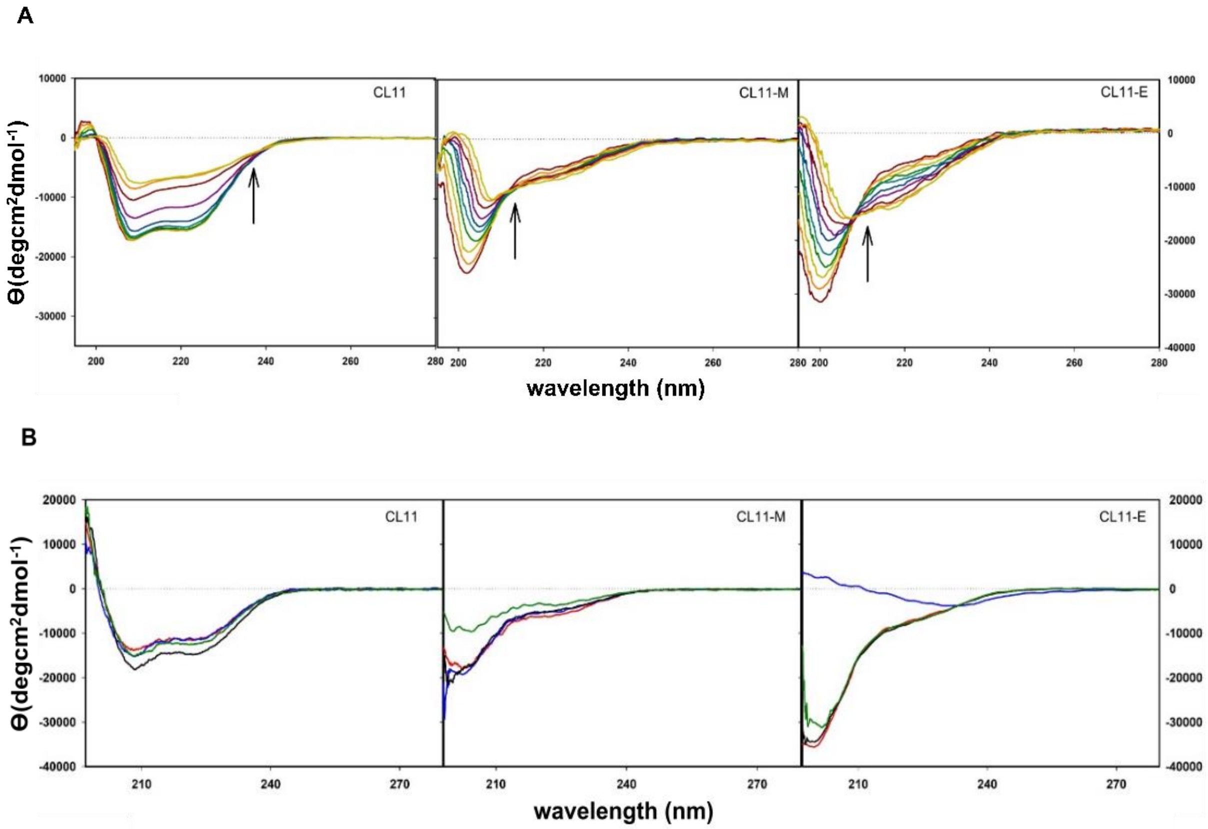

Fig. S3. ECD spectra of protein CL11, CL11-E and CL11-M. (A) ECD spectra of protein CL11, CL11-E and CL11-M in temperature interval 5-95°C (arrows indicate tendency of the spectral changes with temperature increase by 10°C steps). (B) The ECD spectra of CL11, CL11-E and CL11-M in buffer pH 7.9 (black), buffer pH 3.5 (blue), buffer pH 10.5 (green) and buffer pH 7.9 without Mg^{2+} and K^{+} (red).

A

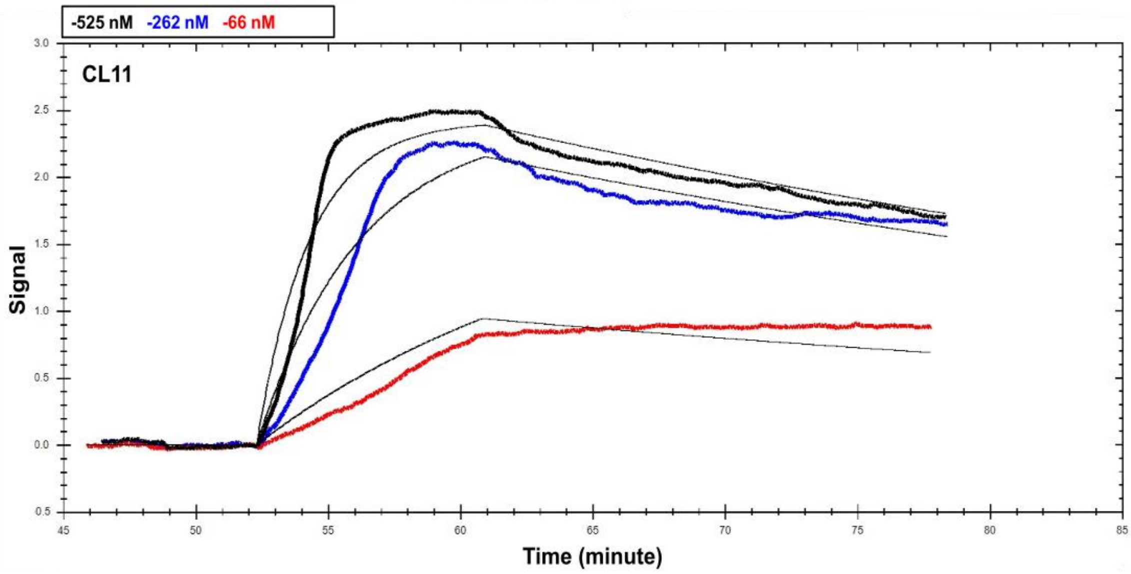
-525 nM -262 nM -66 nM
CL11
Signal
Time (minute)

B

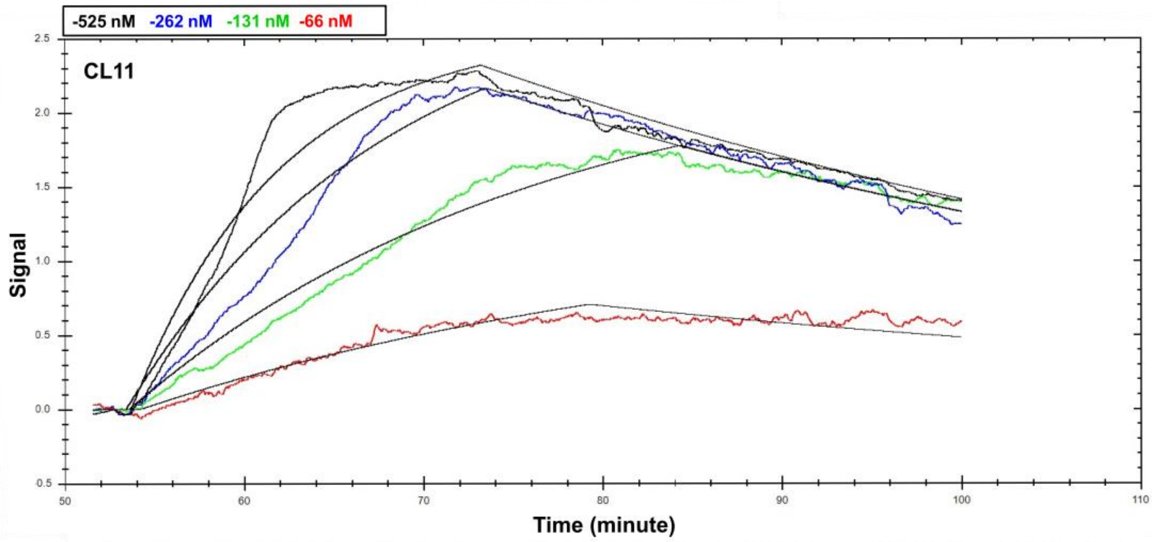
-525 nM -262 nM -131 nM -66 nM
CL11
Signal
Time (minute)

C

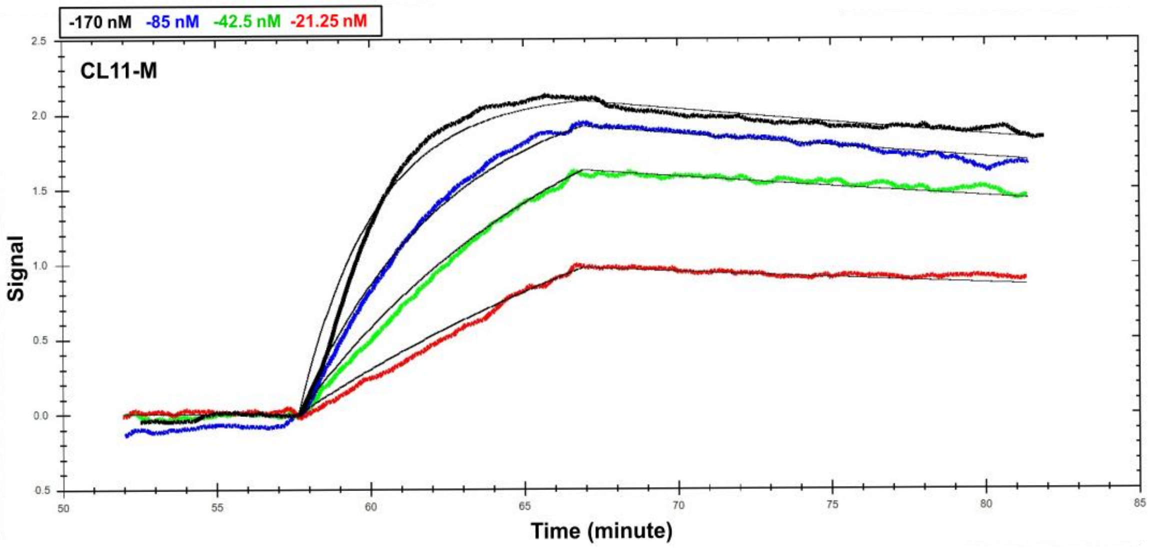
-170 nM -85 nM -42.5 nM -21.25 nM
CL11-M
Signal
Time (minute)

D

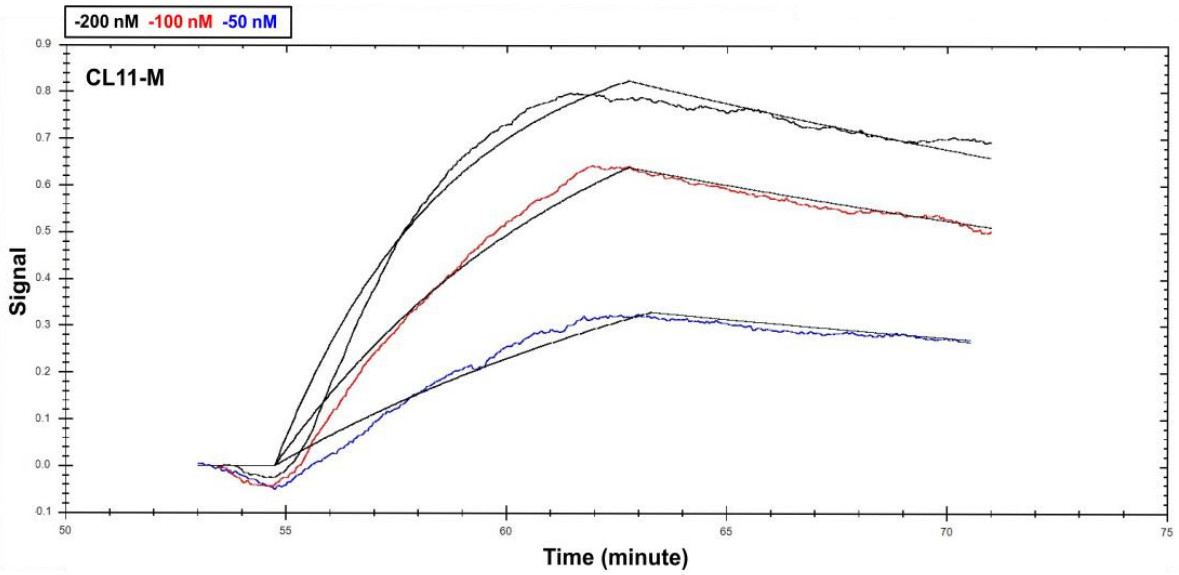
-200 nM -100 nM -50 nM
CL11-M
Signal
Time (minute)
0.9
0.8
0.7
0.6
0.5
0.4
0.3
0.2
0.1
0.0
-0.1
50
55
60
65
70
75

E

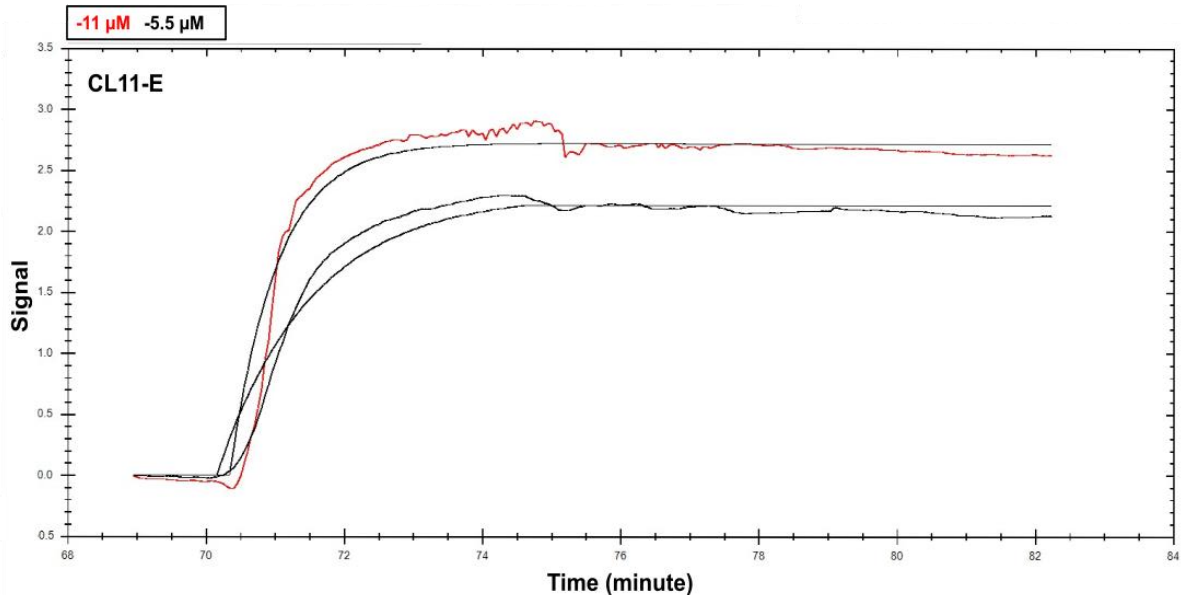
-11 μM -5.5 μM
CL11-E
Signal
Time (minute)
3.5
3.0
2.5
2.0
1.5
1.0
0.5
0.0
0.5
68
70
72
74
76
78
80
82
84

F

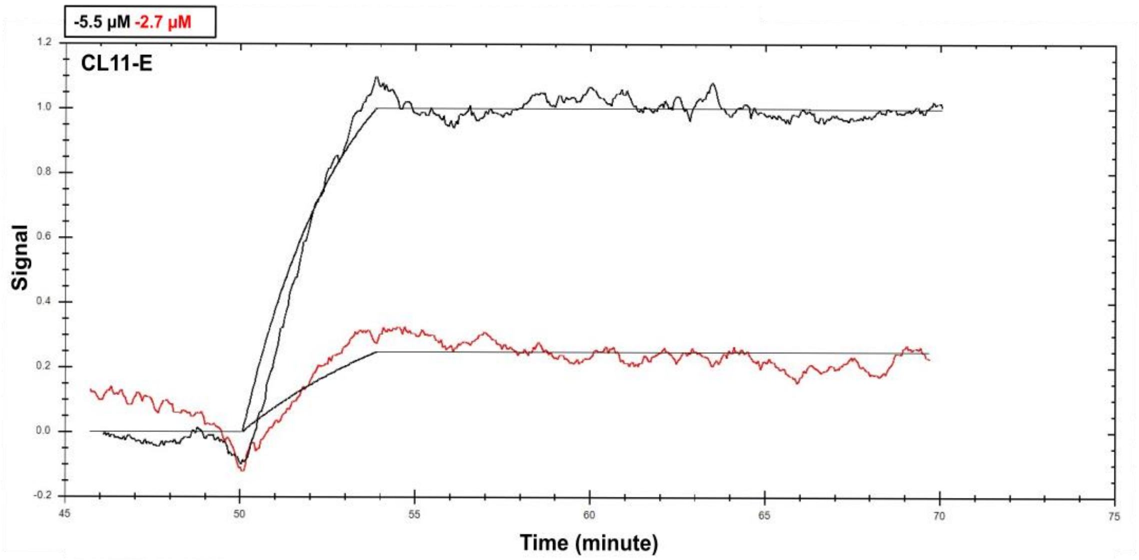

G

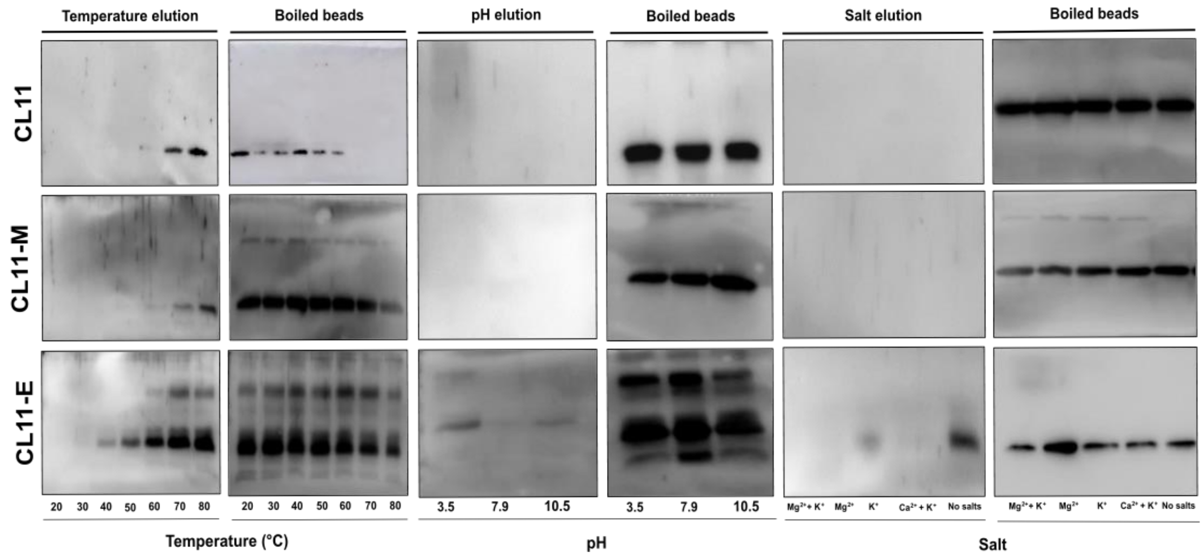

Fig. S4. Complex characterization of the CL11, CL11-M and CL11-E. (A-F) The biotinylated target 58rRNA was immobilized on SPR chips through a neutravidin−biotin interaction. For each protein different concentrations were injected (association phase) and then buffer R with 0.1% Triton X100 was injected (dissociation phase). (G) Uncropped Western Blot of the protein-RNA complex stability at different temperatures, pH and salts presence.

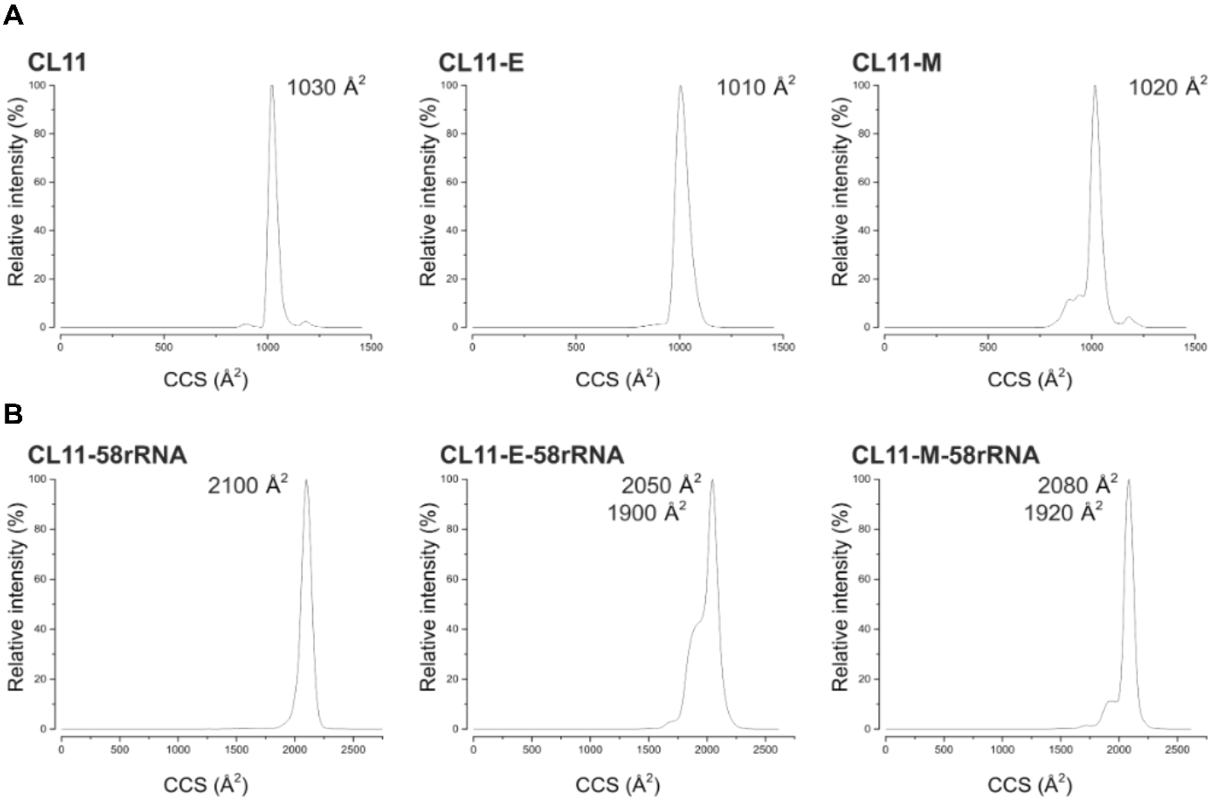

Fig. S5. Native ion mobility - mass spectrometry (IM-MS) measurements. Ion mobility traces for CL11, CL11-E and CL11-M (A) and their complexes with 58rRNA (B). Collision cross sections were calculated for quintuple charge proteins (CL11 1794 *m/z*, CL11-E 1659 *m/z*, CL11-M 1765 *m/z*) and 9^+ charge states of protein-RNA complexes (CL11-58rRNA 3100 *m/z*, CL11-E-58rRNA 3026 *m/z*, CL11-M-58rRNA 3084 *m/z*).

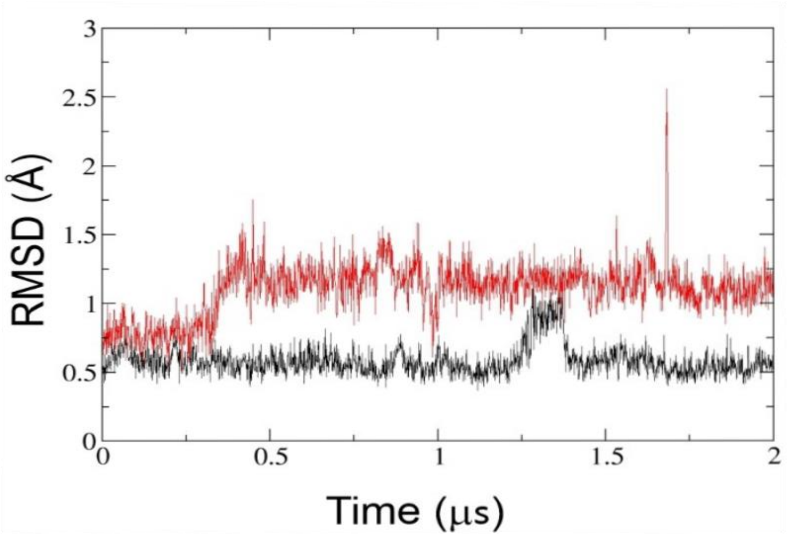

Fig. S6. RMSD of protein secondary structure backbone atoms during 2 μs MD of CL11-58rRNA (black) and CL11-E-58rRNA (red).

Table S1. Total E library enrichments from round 6th to 16th round selection.

Selection round	% of CL11-E	Total reads	CL11-E read count	Protein count
6	0.03	69311	21	63438
9	0.10	87175	91	49735
11	0.54	45019	245	22869
14	6.09	32495	1980	16853
16	9.17	31626	2901	15859

Table S2. Total M library enrichments from round 1st to 10th round selection.

Selection round	% of CL11-M	Total reads	CL11-M read count	Protein count
1	0.03	123778	38	98230
4	0.04	180776	80	94308
10	4.28	137716	5896	59065

Table. S3. Analysis of the secondary structure content of CL11, CL11-M and CL11-E

	**Helix**	**3/10 helix**	**Sheet**	**Turn**	**PP2**	**Unord**
**CL11**	45%	7%	0%	12%	5%	32%
**CL11-M**	6%	8%	12%	16%	11%	47%
**CL11-E**	17%	12%	0%	17%	11%	42%

*(Sreerama and Woody 2000; Sreerama and Woody 2004)

Table. S4A. Direct CL11-58rRNA hydrogen bonding.

Residue	Residue position	Atom	RNA	Nucleotide position	Atom	Occupancy (%)	Backbone (b) Side chain (s)
ALA	9	N	U	10	OP1	99.8	b
LYS	14	NZ	C	14	OP1	71	s
GLY	22	N	G	13	O3'	23.6	b
GLY	22	N	C	14	OP1	100	b
SER	23	O	G	13	O2'	100	b
GLY	24	O	G	13	N2	93.2	b
ASN	27	OD1	C	26	O2'	91.8	s
ASN	27	ND2	A	27	OP1	58.8	s
ARG	28	NH2	C	26	O3'	22.2	s
ARG	28	NH1	C	26	O3'	21.2	s
ARG	28	NH1	A	27	OP1	29.8	s
ARG	28	NH2	A	27	OP1	23.6	s
LYS	46	NZ	U	10	OP2	96.4	s
ASP	49	O	U	8	O2'	52.8	b
ASN	51	ND2	U	8	O2	11.2	s
ASN	51	ND2	U	31	O2	94.8	s
ASN	51	ND2	U	32	O2	56	s
ARG	60	NE	A	30	O3'	34.8	s
ARG	60	NH2	A	30	O3'	26.2	s
ARG	60	NE	U	31	OP1	57.6	s
ARG	60	NH2	U	31	OP1	46	s
ARG	60	NH1	U	31	OP1	37.4	s
MET	61	O	G	9	N2	100	b
GLY	64	O	A	38	N6	97.4	b
THR	65	OG1	U	10	O4	96.8	s
ARG	67	NH1	A	27	O3'	31.4	s
ARG	67	NH2	U	28	OP1	74	s
ARG	67	NH1	U	28	OP1	66.8	s
SER	68	O	G	12	N2	96.2	b
SER	68	OG	G	12	N3	70.4	s

Table. S4B. Direct CL11-E-58rRNA hydrogen bonding

Residue	Residue position	Atom	RNA	Nucleotide position	Atom	Occupancy (%)	Backbone (b) Side chain (s)
GLY	22	N	G	13	O3'	27.2	b
GLY	22	N	C	14	OP1	97	b
SER	23	O	G	13	O2'	100	b
GLY	24	O	G	13	N2	85.6	b
GLU	27	N	C	26	O2'	35.4	b
ALA	52	N	U	31	O2'	38.4	b
SER	61	O	G	9	N2	99.6	b
GLY	64	O	A	38	N6	62.6	b
THR	65	OG1	U	10	O4	95.8	s
SER	68	O	G	12	N2	99	b
SER	68	OG	G	12	O2'	17.4	s
SER	68	OG	G	12	N3	11.2	s
SER	68	OG	G	12	O2'	33.2	s
SER	68	OG	A	27	O2'	31.6	s
SER	68	OG	A	38	N6	12.4	s
SER	68	OG	A	38	N1	36	s
SER	68	OG	A	38	N6	24.8	s
